# Remarkable convergent evolution in specialized parasitic Thecostraca (Crustacea)

**DOI:** 10.1186/1741-7007-7-15

**Published:** 2009-04-17

**Authors:** Marcos Pérez-Losada, Jens T Høeg, Keith A Crandall

**Affiliations:** 1 CIBIO, Centro de Investigação em Biodiversidade e Recursos Genéticos, Universidade do Porto, Campus Agrário de Vairão, Portugal; 2 Comparative Zoology, Department of Biology, University of Copenhagen, Copenhagen, Denmark; 3 Department of Biology and Monte L Bean Life Science Museum, Brigham Young University, Provo, Utah, USA

## Abstract

**Background:**

The Thecostraca are arguably the most morphologically and biologically variable group within the Crustacea, including both suspension feeders (Cirripedia: Thoracica and Acrothoracica) and parasitic forms (Cirripedia: Rhizocephala, Ascothoracida and Facetotecta). Similarities between the metamorphosis found in the Facetotecta and Rhizocephala suggests a common evolutionary origin, but until now no comprehensive study has looked at the basic evolution of these thecostracan groups.

**Results:**

To this end, we collected DNA sequences from three nuclear genes [18S rRNA (2,305), 28S rRNA (2,402), Histone H3 (328)] and 41 larval characters in seven facetotectans, five ascothoracidans, three acrothoracicans, 25 rhizocephalans and 39 thoracicans (ingroup) and 12 Malacostraca and 10 Copepoda (outgroup). Maximum parsimony, maximum likelihood and Bayesian analyses showed the Facetotecta, Ascothoracida and Cirripedia each as monophyletic. The better resolved and highly supported DNA maximum likelihood and morphological-DNA Bayesian analysis trees depicted the main phylogenetic relationships within the Thecostraca as (Facetotecta, (Ascothoracida, (Acrothoracica, (Rhizocephala, Thoracica)))).

**Conclusion:**

Our analyses indicate a convergent evolution of the very similar and highly reduced slug-shaped stages found during metamorphosis of both the Rhizocephala and the Facetotecta. This provides a remarkable case of convergent evolution and implies that the advanced endoparasitic mode of life known from the Rhizocephala and strongly indicated for the Facetotecta had no common origin. Future analyses are needed to determine whether the most recent common ancestor of the Thecostraca was free-living or some primitive form of ectoparasite.

## Background

**Figure 1:**
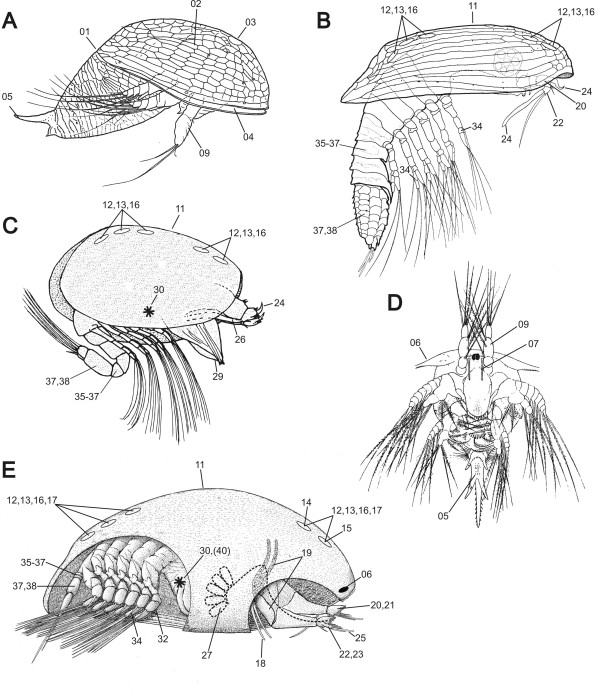
**Thecostracan larvae**. A. Y-nauplius (Facetotecta). B. Y-cyprid (Facetotecta). C. A-cyprid (Ascothoracida). D. Nauplius (Cirripedia), with fronto-lateral horns. E. Cyprid (Cirripedia), with part of the right side carapace cut away to reveal antennules and thorax. The numbers indicate the position of selected characters used in the phylogenetic analysis. A-D redrawn from various sources, E, original.

The Thecostraca, which include the Facetotecta, Ascothoracida and Cirripedia, is a highly variable crustacean group in terms of both morphology and biology [1,2]. This makes them prime models for studying evolutionary adaptations in both morphology and reproductive systems [3]. In fact, the specializations in adult morphology, growth, feeding biology and sexual systems prompted Darwin to study cirripedes, resulting in one of the first 'model organisms' of evolutionary adaptation [4-7]. All thecostracans are sessile as adults and to initiate this phase they have evolved the cypridoid larva, called cyprid in the Cirripedia, a-cyprid in the Ascothoracida and y-cyprid in the Facetotecta (Figure 1). The cypridoid follows after the naupliar phase and has prehensile antennules and natatory thoracopods [8,9]. The Thecostraca include both suspension feeders (Cirripedia: Thoracica and Acrothoracica) and advanced parasitic forms [Rhizocephala (Cirripedia), Ascothoracida and Facetotecta].

The Thoracica (Cirripedia) is highly specialized in having their six pairs of thoracopods modified into a basket of cirri used in suspension feeding [10]. In addition, their body armour consists of a system of mineralized plates. These are not shed at moults but increase gradually in size, which differs from the conventional crustacean mode of growth by a series of cuticular moults [10,11]. Thoracican cirripedes are prime models for studies on intertidal ecology [12-15], larval settlement [16,17], antifouling technology [18] and for testing theories on the evolution of life cycles and reproductive systems [3,19-22]. The Acrothoracica (Cirripedia), though similarly suspension feeders by means of thoracic cirri, lack mineralized plates [23]. Instead, they are symbiotic and inhabit self-excavated burrows in either corals or gastropod shells occupied by hermit crabs.

**Figure 2:**
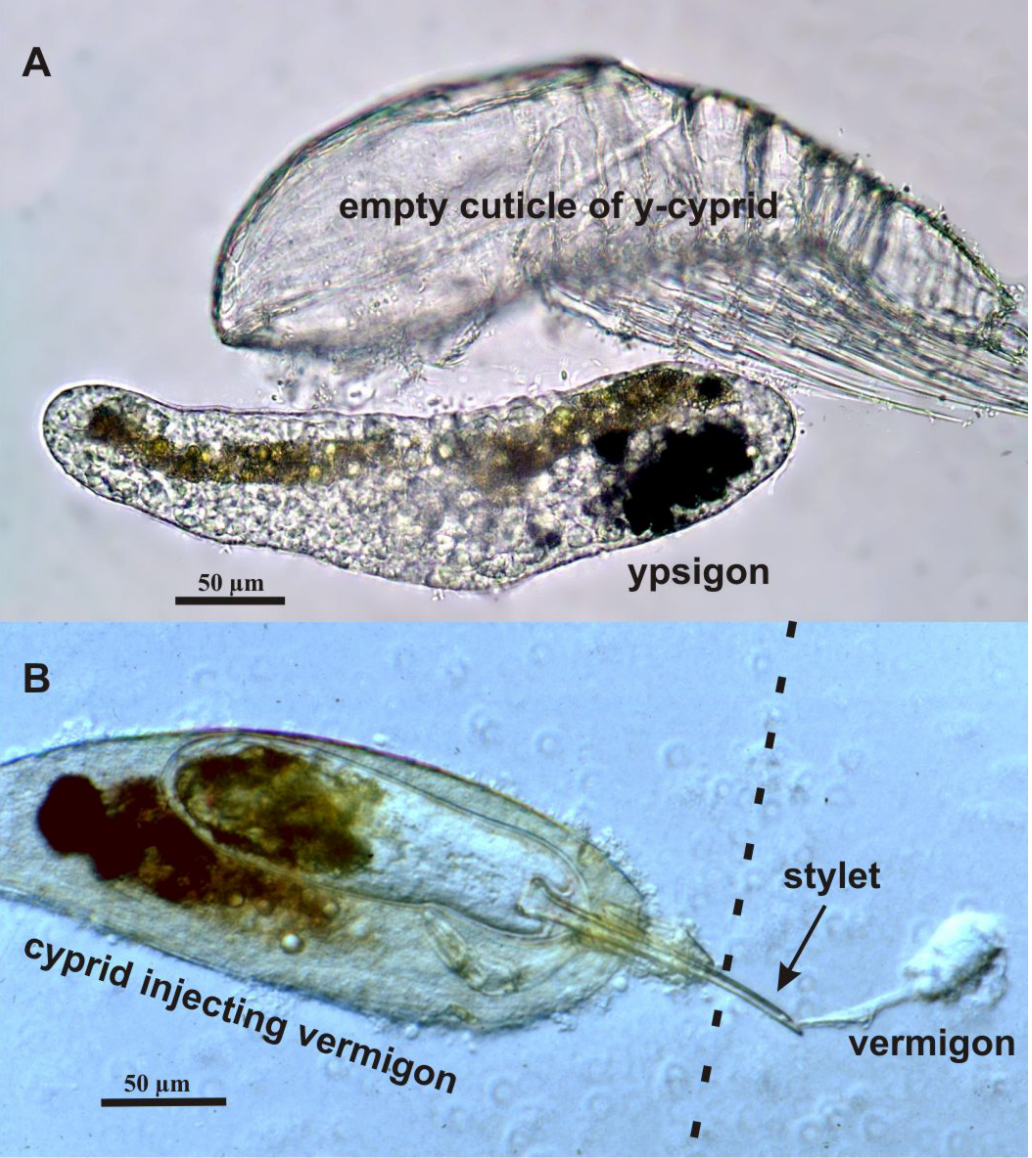
**Metamorphosis in the Facetotecta and Rhizocephala**. A. Y-cyprid (Facetotecta) has metamorphosed into the slug-shaped ypsigon stage, leaving behind the empty cuticle of the cyprid (adapted from [35]). B. A cyprid of *Sacculina carcini *(Rhizocephala) settled on a crab has formed an injection stylet through which it is now injecting the slug-shaped vermigon into the haemocoelic fluid of the host. The specimen was removed *in vivo *from the host cuticle which would have been at the dotted line. Photo by J. Høeg and H. Glenner.

All species of the Rhizocephala (Cirripedia) are parasites on crustaceans, mostly on Decapoda, although they can also infest a range of peracarids, stomatopods and even thoracican barnacles. They are so specialized that they can only be recognized as Crustacea by means of their larvae [24-26]. The adult parasite, consisting of an external reproductive sac and a system of rootlets ramifying inside the host, is simplified to such an extent that it lacks all organs and structures normally used to identify Crustacea and other arthropods [19,27,28]. Rhizocephalans show a highly complex life cycle including their mode of infestation (Figure 2B). When settled, the rhizocephalan cypris larva injects itself through the cuticle of the host crab and into the haemocoel as a so-called vermigon stage that has an exceedingly simplified structure. It is worm shaped and self motile, but consists merely of a very thin epicuticle and four types of cells. There is no trace of segmentation, appendages or any kind of differentiated organs except an epidermis and a lump of cells representing the primordial ovary. The vermigon migrates inside the host until it reaches the site where the adult parasite will grow out its body parts and eventually emerge on the host's exterior [29,30].

The Ascothoracida (Thecostraca) are also parasites and infest either echinoderms or cnidarians. The most plesiomorphic ascothoracids are very little modified and only the piercing mouthparts indicate their ectoparasitic mode of life. More advanced taxa, such as the Dendrogastridae, can have strongly modified endoparasitic adults with long root-like extensions of the body wall, but they always retain body segmentation and appendages [31]. The details of host infection, including how the endoparasitic ascothoracids gain access to their host, have never been observed, but in the ectoparasitic species the adult differs little if at all from the a-cypris larva, indicating that no real metamorphosis takes place [31,32].

The Facetotecta is the most elusive group within the Thecostraca sensu Grygier [8]. First recorded more than 100 years ago [33], they occur as y-larvae in marine plankton throughout the world, but the adult stage has never been found in the wild [34]. However, recently, Glenner *et al. *[35] induced facetotectan y-cyprids to metamorphose *in vitro *(Figure 2A). They moulted into a slug-shaped stage called the ypsigon, which exhibits many similarities with the rhizocephalan vermigon. From this comparison Glenner *et al. *[35] concluded that adult facetotectans are parasites and that the ypsigon initiates an internal (endoparasitic) phase of their life cycle. The similarity between the ypsigon and vermigon stages raises the question of the phylogenetic relationship between the Facetotecta and the Rhizocephala and whether their advanced modes of parasitism have a common evolutionary origin. However, a sister relationship between the Facetotecta and the Rhizocephala goes against the classic idea of a monophyletic Cirripedia, which is supported by the presence of the so-called fronto-lateral horns in the nauplii [1,26]. The facetotectans become even more interesting by the observation that their larvae can be both abundant and diverse, such as in Okinawan waters where more than 40 putative species occur at a single study site, indicating that a similar number of undescribed parasites occur in the nearby coral reef habitat [35,36].

Historically, the term Cirripedia was used rather loosely, often incorporating the Ascothoracida, while the facetotectan y-larvae had no systematic affiliation. Since Grygier [8], the subclass Thecostraca, in systematic terms, has comprised the Facetotecta (y-larvae), the Ascothoracida and the Cirripedia, while the Cirripedia consists of the Acrothoracica, the Rhizocephala and the Thoracica [37]. Grygier [8] based his system on a formal numerical cladistic analysis, but having at his disposal only a limited set of morphological characters he failed to resolve relations among the three thecostracan or among the three cirripede clades. In the last 10 years, cirripede phylogeny has been studied rather intensively based on molecular datasets. It now appears that the Rhizocephala are monophyletic [38], as suggested by the 18S rRNA locus, and some insight has also been gained in the intrinsic relationships of the Thoracica using nuclear (18S rRNA, 28S rRNA and H3) and mitochondrial (12S rRNA and 16S rRNA) loci [39,40]. For the Thecostraca in general, Pérez-Losada *et al. *[41] used 18S rRNA sequences to argue that the Facetotecta is the sister group to an Ascothoracida + Cirripedia clade, but this was based on only a single facetotectan species. There is accordingly an urgent need to re-examine the phylogeny of all Thecostraca using a wider taxon sampling across the five major taxa. Moreover, since Grygier [8], no study has presented a character matrix that covers morphological features across all Thecostraca. Such a matrix must necessarily be based on larval characters, because post-larval Facetotecta and Rhizocephala are so reduced that no features can be compared with other groups [25]. Even a comparison between the dissimilar adults of the Ascothoracida, Acrothoracica and Thoracica meets with severe difficulties [42].

Here we present a large-scale analysis combining a morphological dataset of 41 larval characters with sequence data from three nuclear genes and with broad taxon sampling within all the major thecostracan groups. Our purpose was to obtain a well-supported phylogeny for testing how often parasitism evolved within the subclass and whether homology exists between the advanced metamorphosis found in both the Facetotecta and Rhizocephala. We generated a robust phylogenetic tree where the main Thecostraca relationships were depicted as (Facetotecta, (Ascothoracida, (Acrothoracica, (Rhizocephala, Thoracica)))). This topology suggests that the very similar and highly advanced mode of metamorphosis found in the Facetotecta and the Rhizocephala evolved independently, providing a remarkable case of convergent evolution into extremely specialized endoparasitism.

## Results

**Figure 3:**
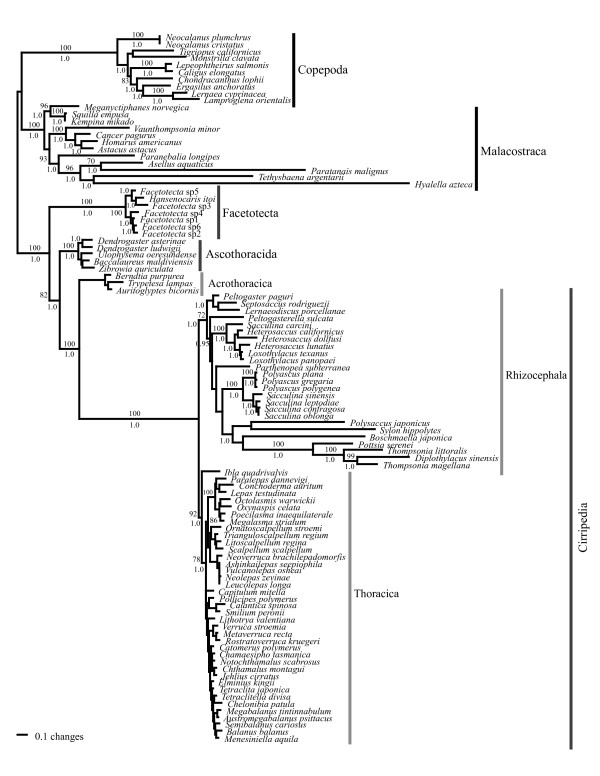
**DNA maximum likelihood and morphological-DNA Bayesian trees**. Branch lengths are shown proportional to the amount of change along the branches of the maximum likelihood tree. Bootstrap proportions (if ≥70%) and clade posterior probabilities (if ≥95%) are shown for each node. Support for some shallow clades is not shown.

Both the DNA maximum likelihood (ML) and morphological-DNA Bayesian (BMCMC) analyses resulted in trees where the Facetotecta, Ascothoracida and Cirripedia were each monophyletic (Figure 3). Those trees were also well resolved and supported, and they only differed from each other in minor details. The Facetotecta branched off basally as the sister group to all remaining Thecostraca. Within the latter clade, the Ascothoracida was sister to a monophyletic Cirripedia comprising the Acrothoracica, Rhizocephala and Thoracica. Within the Ascothoracida, neither the order Dendrogastrida nor the family Dendrogastridae were monophyletic in our trees, because *Ulophysema *had a basal position separated from *Dendrogaster *by *Zibrowia *and *Baccalaureus*, both of which are currently classified in the order Laurida; nonetheless, this clade had no significant support [bootstrap proportions (bp) <70% and posterior probabilities (p*P*) < 0.95]. Moreover, a monophyletic Dendrogastrida hypothesis had a p*P *< 0.019, but was not rejected by the S-H test. Within the Cirripedia, the three main groups were each monophyletic with the Acrothoracica depicted as sister to a Rhizocephala + Thoracica clade. Within the Rhizocephala, neither the ML nor the Bayesian trees showed the Kentrogonida or Akentrogonida as monophyletic. The reason is that *Sylon hippolytes*, which belongs to the Kentrogonida, is sister to *Polysaccus japonicus*, which belongs to the Akentrogonida and this latter clade has a p*P *= 1.0. A Kentrogonida + Akentrogonida clade was rejected by both the Bayesian (p*P *< 0.001) and S-H (*P *< 0.05) tests. Within the Thoracica, the ML and Bayesian trees had the same general topology as in Pérez-Losada *et al. *[39,40], although some taxa fell in a different position in our new trees (for example, *Smilium *and *Calantica*). Nonetheless, the current analyses and morphological data were never meant to address the intrinsic relationships within the Thoracica; hence, we refer the reader to our latest study [39] for a more robust analysis of the group.

**Figure 4:**
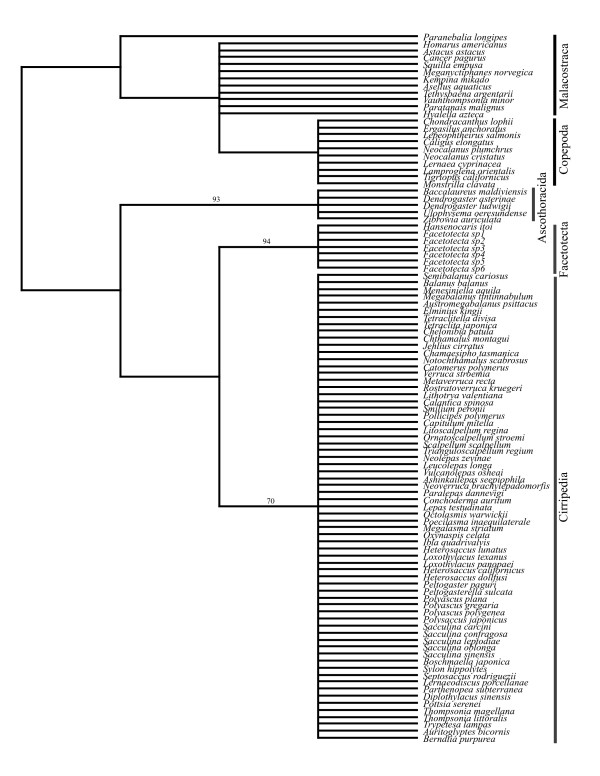
**Morphological strict-consensus maximum parsimony tree**. Bootstrap proportions (if ≥70%) are shown for each node.

Our maximum parsimony (MP) analysis of the morphological characters alone yielded a strict consensus tree (*L *= 55) recovering the Facetotecta, Ascothoracida and Cirripedia as monophyletic, although no resolution or support was observed within clades (Figure 4). This tree also showed a Facetotecta-Cirripedia sister relationship which is supported by seven characters: nauplius with furcal setae (character 5; Additional file 1, Appendix S1), [43-45], antennular segment number expressed in entire life cycle (8), naupliar antennular segments (9), a hand, hoof or bell-shaped semi-distal antennular segment in the cypridoid larva (20), mouthparts and gut (29), reduction of thoracopodal musculature (31) and profound metamorphosis after cypridoid stage (39). This highlights the similarity between Facetotecta and Cirripedia due to convergence, as compared with our better-resolved ML and BMCMC trees where these clades do not form sister groups. Obviously, in the combined BMCMC analysis, the signal from the morphological data is overwhelmed by the DNA data, which has many more informative characters with stronger phylogenetic signal. When the same larval data were mapped on the ML tree or included in the BMCMC analysis, only three characters supported an Ascothoracida-Cirripedia clade: frontal filaments in nauplius (7), reduction of distal antennular musculature in cypridoid larva (26) and postoral adductor muscle (30). Moreover, 11, 3 and 5 apomorphies supported the monophyly of Cirripedia, Ascothoracida and Facetotecta, respectively.

## Discussion

### Phylogenetic relationships

All our molecular and combined analyses (Figure 3) supported the Facetotecta, Ascothoracida, Acrothoracica, Rhizocephala and Thoracica as monophyletic taxa with high confidence. For the Rhizocephala, the molecular analysis by Glenner and Hebsgaard [38] has already provided strong support for the monophyly of this taxon, but their study did not include the Facetotecta. For the Thoracica, previous analyses had either very limited taxon sampling [46-49] or assumed the taxon to be monophyletic [39,40]. Our analysis is therefore the first to test the monophyly of the above mentioned taxa using a comprehensive taxon sampling covering all thecostracan orders.

Morphological evidence alone could not resolve the phylogenetic position of the Facetotecta [8]. In our ML and BMCMC analyses (Figure 3), the Facetotecta was depicted at the base of the thecostracan tree, which confirms previous results by Pérez-Losada *et al. *[41] based on a single 18S rRNA sequence from the facetotectan *Hansenocaris itoi*. The Facetotecta is the only crustacean taxon where the taxonomy is based exclusively on larval forms [34]. All Facetotecta described to date belong to the genus *Hansenocaris*, although undoubtedly this covers a more extensive taxonomic diversity [35]. Kolbasov *et al. *[50] provided a key to all known facetotectan species, whether formally described or not, and they list a host of morphological characters that could be used for analysing the intrinsic phylogeny of the taxon. Our molecular analysis (Figure 3) of seven facetotectans had full resolution and high support for many nodes, indicating that the gene regions used here will also be useful for resolving evolutionary relationships within the Facetotecta. This, and the many features in larval morphology, bodes well for a future phylogenetically based taxonomy of the group.

Within the Ascothoracida, our analyses did not recover a monophyletic Dendrogastridae. Similarly, Kolbasov *et al. *[50] using lattice organ morphology at a detail not coded for here, also questioned the validity of this family. Nonetheless, taxon sampling is still limited within the subclass, and it must be kept in mind that the present classification of the Ascothoracida was never claimed to be cladistically based [51].

Within the Rhizocephala, our ML and Bayesian trees largely agreed with the recent analysis in Glenner and Hebsgaard [38], although we did not retrieve a monophyletic Akentrogonida. Both the ML and Bayesian analyses confirmed previous claims that the Rhizocephala and the Thoracica are sister groups [41], while the Acrothoracica diverged at the base of the Cirripedia. Two evolutionary scenarios can account for this: (1), an acrothoracican-type ancestor has led equally to both the Thoracica and the Rhizocephala; (2) a thoracican-like ancestor of both lineages became modified in both the Acrothoracica and the Rhizocephala, while its morphology remains reflected in the most plesiomorphic extant thoracicans such as the Iblomorpha [39].

Grygier [8] listed a number of putative apomorphies for the Thecostraca, mostly pertaining to the presence of a cypridoid settlement stage with prehensile antennules. To his list we can now add the presence of five pairs of chemosensory lattice organs on the carapace of the cypridoid larva [50,52-54]. These structures have not been found in any other crustaceans.

### Larval morphology

Larval characters are the only morphological traits that can be coded for all thecostracan taxa, but only Grygier [8] has previously presented a formalized matrix based on these features. Although our morphological dataset failed to resolve basic thecostracan radiation in the MP analysis, we did identify apomorphies in support of the monophyly of the three major groups. For the Cirripedia, one of the 11 morphological apomorphies is the classical presence of fronto-lateral horns in the nauplii, which was the first character by which the Rhizocephala could be recognized as belonging to the Cirripedia [1,10]. The remaining 10 apomorphies all concern the cyprid, which is instrumental in enabling the complex and efficient mechanism of substratum location and attachment seen in all cirripedes irrespective of the immense differences in adult morphology and biology. As previously predicted [9,26] these cyprid specializations, notably in the sensory and locomotory apparatus, are important in explaining the immense success of the Cirripedia compared with the other Thecostraca. When facetotectan y-cyprids and ascothoracidan a-cyprids become better analyzed at a level comparable to that of the cirripede cyprid (e.g., [55]), it is very likely that additional apomorphies for the Cirripedia will be revealed.

### Parasitism in the Thecostraca

The Facetotecta, Ascothoracida and Rhizocephala are all parasitic and this raises the question whether parasitism in these taxa evolved independently or had some level of common ancestry. Both our outgroups contain parasitic forms, but these are all secondarily derived with high certainty. In the Malacostraca, parasites are found only in highly derived taxa such as the Amphipoda and especially the Isopoda. Both isopods and amphipods are nested deep within the Malacostraca and the parasites again deep within the two orders [56-59]. In the Copepoda all available phylogenies, notably the one by Huys and Boxshall [60], place the several parasitic groups well derived within the taxon. We can therefore assume a free-living ancestor at the base of both outgroups, which suggests that the ancestor of the Malacostraca, Copepoda and Thecostraca was free-living too. Making this assumption, our phylogeny indicates a shift from free-living to parasite at the base of the Thecostraca, as the Facetotecta (parasite) is the first group to branch off. However, our morphological dataset was not constructed to solve this issue, since it relies on larval characters only. Moreover, coding parasitism as a single character is a gross oversimplification, obscuring the different adaptations to this mode of life within the Thecostraca. While detailed similarities exist between Facetotecta and Rhizocephala, neither of these taxa shows any similarity to the Ascothoracida in their mode of parasitism. In the most primitive Ascothoracida there is no metamorphosis, and the adult parasite is very similar to the settling a-cypris larva. This contrasts with the profound and remarkably similar metamorphosis known from both the Facetotecta and Rhizocephala. In rhizocephalans the settled cyprid metamorphoses into a vermigon, a highly reduced stage that is injected into the haemocoelic system of the host crab and initiates the endoparasitic phase of the life cycle (Figure 2B) [29,30,61]. Recently, Glenner *et al. *[35] found that facetotectan y-cyprids metamorphose into a very comparable stage called the ypsigon (Figure 2A). Both the vermigon and the ypsigon are slug-shaped, unsegmented, without appendages and have an extremely simplified internal structure that includes only a handful of cell types (Figures 1 and 2). Based on these observations, Glenner *et al. *[35] concluded that the adult Facetotecta are parasitic in unknown hosts and, like the Rhizocephala, with an initial endoparasitic phase in the life cycle. Our ML and BMCMC analyses (Figure 3) found no close relation between the Facetotecta and Rhizocephala, and such a relation was rejected by both the S-H and Bayesian tests (*P *and p*P *< 0.001). If mapped onto our ML or BMCMC trees, the presence of a slug shaped, unsegmented stage (vermigon, ypsigon) appears to have evolved independently in the Facetotecta and the Rhizocephala. This remarkable convergence demonstrates the flexibility of the thecostracan body plan and the morphological diversity crustaceans can achieve when under selection for parasitism. As complementary work to the analysis presented here we would, in the future, like to include the parasitic Tantulocarida, from which we did not have material for molecular analysis [37,62,63]. This class of Crustacea is often assumed to be the closest relative to the Thecostraca. It would therefore be very interesting to examine if the highly advanced parasitism seen in this group is homologous to any of the several modes of parasitism found in the Thecostraca.

## Conclusion

We conclude that the Facetotecta is a monophyletic taxon with a sister relationship to a clade consisting of the Ascothoracida and Cirripedia. Within the latter, the Acrothoracica is sister to a Rhizocephala + Thoracica clade. Facetotecta, Ascothoracida and Cirripedia are each well characterized by apomorphies in larval morphology. The very similar ypsigon and vermigon stages arose independently in the Facetotecta and the Rhizocephala and provide a remarkable case of convergent evolution. Future analyses with an enlarged morphological database and more taxa must elucidate whether the ancestors to the Thecostraca were free-living or some form of primitive ectoparasites.

## Methods

### Taxon sampling

We sampled 79 thecostracans including Facetotecta (seven species), Ascothoracida (five spp.), and Cirripedia (67 spp.): Acrothoracica (three spp.), Rhizocephala (25 spp.) and Thoracica (39 spp.). While there is no consensus on large-scale phylogeny of the Crustacea, the most recent and comprehensive phylogenetic analysis of arthropod relationships [64] based on 62 loci (41 Kb) showed that the Malacostraca is the sister clade to the Thecostraca and the Copepoda is their closest relative, confirming previous phylogenetic results by Regier *et al. *[65] and Mallat and Giribet [66]. We, therefore, used 12 malacostracans and 10 copepods (Additional file 1, Table S1) to root the Thecostraca tree. All Facetotecta, except *Hansenocaris itoi*, were sampled as nauplii and reared to the y-cypris stage at the Sesoko Marine Station, Okinawa, Japan, as in Glenner *et al. *[35]. DNA was extracted from samples of 10–20 y-cyprids pooled from distinct types of larvae. These types, representing still-undescribed species, were distinguished based on the morphology of moulted skins of the last nauplius V instars that developed into the y-cyprids (see [35]). The most recent classification of the Crustacea by Martin and Davis [37] lists a total of eight orders within the three thecostracan infraclasses (Facetotecta, Ascothoracida and Cirripedia), and all of these are represented in this study (see Additional file 1, Table S1; no subdivision of the infraclass Facetotecta has been attempted yet). We, therefore, think that this represents a reasonable sampling for studying basic evolutionary relationships within the Thecostraca. Specimens were preserved in 70% EtOH and are housed in the crustacean collection at the Monte L. Bean Life Science Museum, Brigham Young University.

### DNA extraction, PCR and sequencing

DNA extraction, amplification, and sequencing were performed as described in Pérez-Losada *et al. *[39,40]. Since this study attempts to solve the evolution of backbone lineages in the thecostracan tree, we selected the three more informative genes in Pérez-Losada *et al. *[40] for inferring deep relationships: 18S rRNA (2,305 bp), 28S rRNA (2,402 bp), and histone H3 (328 bp). As demonstrated in Pérez-Losada *et al*. [39], these genes have proven to be very informative at this level. Here we have generated 39 new sequences (18S rRNA = 13 sequences, 28S rRNA = 12, and H3 = 14), which have been deposited in GenBank under the accession numbers FJ751865–FJ751903 (Additional file 1, Table S1).

### Morphological data

We scored a total of 41 characters in larval morphology taken from the nauplii (10), the cypridoid larvae (28) and from general development (3) (Additional file 1, Appendices S1 and S2). There is no previous morphology-based analysis of the phylogeny of the Ascothoracida or the Acrothoracica (Cirripedia), and for the Rhizocephala (Cirripedia) the morphological matrix in Høeg and Lützen [67] concerned only the order Akentrogonida. For the Thoracica (Cirripedia), most previously used characters were derived from the sessile adults [10,39,40,42,68], but since adult Facetotecta are unknown and adult Rhizocephala are singularly specialized for a parasitic mode of life, any morphology based matrix of all Thecostraca must be based on larval characters only. Our matrix is based on Grygier [8,69], but we have increased the list of characters and revised definitions and scorings. Recent advances in the understanding of the ultrastructure of thecostracan larvae have yielded many new traits, especially concerning the cypridoid stage [9,50,53,70-73]. On the other hand, we were compelled to omit some of the potentially important characters of Grygier [8]. He coded the Facetotecta, Ascothoracida and Cirripedia by reconstructing their supposed ground patterns, but we used an exemplar approach, and this illustrated that accurate information on character states was lacking for many species. We therefore used '?' in all cases where the character state was unknown for a species, even if it is supposedly placed deep within a taxon with an apparently invariable morphology for the trait in question. Unexpected character states such as the occurrence of 'crest in a trough'-type lattice organs (character 13) in some *Chthamalus*, which are nested deep within the Thoracica, shows that this is a prudent approach.

### Phylogenetic analyses

Nucleotide sequences were aligned using MAFFT v5.7 [74] as indicated in Pérez-Losada *et al. *[39] for each of the three thecostracan infraclasses. Final assembling of each aligned group was performed using profile alignment generating a final dataset of 6,244 sites. Uncertainty in our 18S and 28S alignments was identified using GBlocks v0.91b [75]. No questionable regions were observed in the entire H3 alignment. GBlocks parameters (minimum number of sequences for a conserved and a flank position, maximum number of contiguous non-conserved positions, minimum length of a block and allowed gap positions) were set up as 51-85-8-10-all and 38-63-8-10-all for 18S and 28S, respectively. These settings generated a conserved alignment of 3,437 sites. Long and short (after GBlocks) aligned data sets were initially analyzed in RAxML [76] and almost identical ML trees were obtained. Therefore, in all the subsequent analyses performed here we used the long aligned dataset (6,244 sites). Congruence among gene regions was addressed using the Wiens [77] protocol. Separate bootstrap ML analyses [78] were conducted for each of the three genes using RAxML to detect potential areas of strongly supported incongruence as indicated by conflicting nodes with bp ≥70%. No such areas of incongruence were observed in our alignment.

Morphological and DNA sequence data were analyzed under different phylogenetic approaches. Morphological data alone were analyzed using MP as implemented in PAUP* v4b10 [79]. We performed MP heuristic searches using 100 random addition replicates and tree bisection and reconnection branch swapping. A maxtree limit of 1,000 trees per replicate was enforced in each analysis. DNA phylogenies were inferred using the ML approach implemented in RAxML with 100 randomized MP starting trees. Morphological-DNA phylogenies were inferred using Bayesian methods coupled with Markov chain Monte Carlo (BMCMC) inference, as implemented in MrBayes v3.04b [80]. DNA mixed model analyses were performed under both ML and BMCMC procedures. DNA model selection followed the procedure outlined by Posada and Buckley [81] as implemented in ModelTest v3.6 [82]. The GTR+Γ+I model [83] was selected for all gene regions. Within the BMCMC approach, morphological data were analyzed using the 'standard' model. This model is based on the ideas originally presented by Lewis [84]. Essentially, the model is analogous to a JC model [85] (equal substitution rates) except that it has a variable number of states (two in our case). Four independent BMCMC analyses were run in MrBayes with each consisting of four chains. Each Markov chain was started from a random tree and run for 10^7 ^cycles, sampling every 1,000^th ^generation. Model parameters were unlinked, treated as unknown variables with uniform default priors and estimated as part of the analysis. Convergence and mixing were monitored using Tracer v1.4 [86]. All sample points prior to reaching stationary were discarded as burn-in. The p*P *for individual clades obtained from separate analyses were compared for congruence and then combined and summarized on a 95% majority-rule consensus tree [87,88].

Clade support under the MP and ML approaches was assessed using the nonparametric bootstrap procedure [89] with 1,000 bootstrap replicates and one random addition per replicate. Confidence in our best hypotheses of phylogenetic relationships was tested by first creating alternative hypotheses in MacClade [90] as indicated in Pérez-Losada *et al. *[40] and then comparing them under both likelihood and Bayesian frameworks. Likelihood topological tests were conducted using the Shimodaira and Hasegawa (S-H) [91] test as implemented in PAUP*. Ten thousand replicates were performed for every topology test resampling the partial likelihoods for each site (RELL model). Bayesian topological tests were performed as described in Huelsenbeck *et al. *[88].

## Authors' contributions

MP-L and KAC designed the molecular data collection scheme and data analysis protocols. MP-L collected the molecular data, generated the sequence alignment and carried out the molecular and some of the morphological analyses. JTH developed the morphological matrix and carried out some of the morphological analyses. All authors participated in the design of the study, obtaining funding for the project, and drafting of the manuscript. All authors have read and approved the final manuscript.

## Supplementary Material

Additional file 1 **Supplementary material**. Supplementary material containing Table S1 and Appendices S1 and S2. Click here for file
