# Supplementary material for: Remarkable convergent evolution in specialized parasitic Thecostraca (Crustacea)
Source: BMC Biol. 2009 Apr 17;7:15. doi: 10.1186/1741-7007-7-15 (PMC2678073; doi:10.1186/1741-7007-7-15)
Supplement: Additional file 1 — Supplementary material. Supplementary material containing Table S1 and Appendices S1 and S2. [file 1741-7007-7-15-S1.doc]

**Additional material**

**Table 1.** Facetotecta, Ascothoracida and Cirripedia (Thecostraca) and Malacostraca and Copepoda (outgroup) included in this study with associated GenBank accession numbers. Bold font style is used for the sequences generated in this study.

| Species | Location | 18SrRNA | 28SrRNA | H3 |
| --- | --- | --- | --- | --- |
| FACETOTECTA |  |  |  |  |
| *Hansenocaris itoi* Kolbasov & Høeg | White Sea | AF439393 | **FJ751865** | **FJ751890** |
| Facetotecta sp. 1 | Okinawa, Japan | **FJ751877** | **FJ751866** | **FJ751891** |
| Facetotecta sp. 2 | Okinawa, Japan | **FJ751878** | **FJ751867** | **FJ751892** |
| Facetotecta sp. 3 | Okinawa, Japan | **FJ751879** | **FJ751868** | **FJ751893** |
| Facetotecta sp. 4 | Okinawa, Japan | **FJ751880** | **FJ751869** | **FJ751894** |
| Facetotecta sp. 5 | Okinawa, Japan | **FJ751881** | **FJ751870** | **FJ751895** |
| Facetotecta sp. 6 | Okinawa, Japan | **FJ751882** | **FJ751871** | **FJ751896** |
| ASCOTHORACIDA |  |  |  |  |
| LAURIDA |  |  |  |  |
| *Baccalaureus maldivensis* Pyefinch | Eilat, Red Sea | **FJ751883** | **FJ751872** | **FJ751897** |
| *Zibrowia auriculata* Grygier | Okinawa, Japan | **FJ751884** | **FJ751873** | **FJ751898** |
| DENDROGASTRIDA |  |  |  |  |
| *Dendrogaster asterinae* Achituv | Sharm el Sheick, Red Sea | **FJ751885** | **FJ751874** | **FJ751899** |
| *Dendrogaster ludwigii* Le Roi | Okinawa, Japan | **FJ751886** | - | **FJ751900** |
| *Ulophysema oeresundense* Brattstrøm | GenBank | L26521 | - | - |
| CIRRIPEDIA |  |  |  |  |
| ACROTHORACICA |  |  |  |  |
| PYGOPHORA |  |  |  |  |
| *Berndtia purpurea* Utinomi | GenBank | L26511 | - | - |
| *Auritoglyptes bicornis* (Aurivillius) | Thailand | **FJ751887** | **FJ751875** | **FJ751901** |
| APYGOPHORA |  |  |  |  |
| *Trypetesa lampas* (Hancock) | Sweden | **FJ751888** | **FJ751876** | **FJ751902** |
| RHIZOCEPHALA |  |  |  |  |
| KENTROGONIDA |  |  |  |  |
| *Heterosaccus californicus* George | California, USA | AY520657 | AY520623 | AY520725 |
| *Heterosaccus dollfusi* Boschma | Israel | EU082413 | EU082333 | EU082373 |
| *Heterosaccus lunatus* Phillips | Queensland, Australia | EU082414 | EU082334 | EU082374 |
| *Lernaeodiscus porcellanae* Müller | GenBank | DQ826569 | - | - |
| *Loxothylacus panopaei* (Gissler) | GenBank | AY265364 | - | - |
| *Loxothylacus texanus* Boschma | GenBank | L26517 | - | - |
| *Polyascus gregaria* (Okada & Miyashita) | GenBank | AY265363 | - | - |
| *Parthenopea subterranea* Kossmann | GenBank | DQ826566 | - | - |
| *Polyascus plana* (Boschma) | GenBank | AY265368 | - | - |
| *Peltogaster paguri* (Rathke) | Sweden | EU082415 | EU082335 | EU082375 |
| *Peltogasterella sulcata* (Lilljeborg) | Sweden | EU082416 | EU082336 | EU082376 |
| *Polyascus polygenea* (Lützen & Takahashi) | GenBank | AY265362 | - | - |
| *Sacculina carcini* Thompson | Sweden | AY520656 | AY520622 | AY520724 |
| *Sacculina confragosa* Boschma | GenBank | AY265361 | - | - |
| *Sacculina leptodiae* Guerin-Ganivet | GenBank | AY265365 | - | - |
| *Sacculina oblonga* Lützen & Yamaguchi | GenBank | AY265367 | - | - |
| *Sacculina sinensis* Boschma | GenBank | AY265360 | - | - |
| *Septosaccus rodriguezii* (Fraisse) | GenBank | DQ826571 | - | - |
| *Sylon hippolytes* Sars | GenBank | DQ826564 | - | - |
| AKENTROGONIDA |  |  |  |  |
| *Boschmaella japonica* Deichmann & Høeg | GenBank | AY265369 | - | - |
| *Diplothylacus sinensis* (Keppen) | GenBank | DQ826568 | - | - |
| *Polysaccus japonicus* Høeg & Lützen | GenBank | DQ826565 | - | - |
| *Pottsia serenei* Lützen & Du | GenBank | DQ826567 | - | - |
| *Thompsonia littoralis* Lützen & Jespersen | GenBank | DQ826573 | - | - |
| *Thompsonia magellana* Høeg & Lützen | Beagle Island, Argentina | **FJ751889** | - | **FJ751903** |
| THORACICA |  |  |  |  |
| PEDUNCULATA |  |  |  |  |
| *Ashinkailepas seepiophila* Yamaguchi, Newman  & Hashimoto | Hatsushima Island, Japan | EU082395 | EU082314 | EU082354 |
| *Calantica spinosa* (Quoy & Gaimard) | Otago, New Zealand | EU082384 | EU082303 | EU082344 |
| *Capitulum mitella* (Linnaeus) | Japan | AY520652 | AY520618 | AY520720 |
| *Conchoderma auritum* (Linnaeus) | Japan | EU082401 | EU082320 | EU082360 |
| *Ibla quadrivalvis* (Cuvier) | Tasmania, Australia | AY520655 | AY520621 | AY520723 |
| *Lepas testudinata* Aurivillius | Cottesloe, Australia | EU082406 | EU082325 | EU082365 |
| *Leucolepas longa* Southward & Jones | S Edison Field, W Pacific Ocean | EU082392 | EU082311 | EU082351 |
| *Lithotrya valentiana* (Gray) | Gulf of Aqaba, Egypt | EU082382 | EU082301 | EU082342 |
| *Litoscalpellum regina* (Pilsbry) | Gulf of Mexico | AY520653 | AY520619 | AY520721 |
| *Megalasma striatum* Hoek | Nansei Islands, Japan | EU082411 | EU082330 | EU082370 |
| *Neolepas zevinae* Newman | East Pacific Rise | EU082391 | EU082310 | EU082350 |
| Octolasmis warwickii (Gray) | Moreton Bay, Australia | EU082409 | EU082328 | EU082368 |
| *Ornatoscalpellum stroemi* (Sars) | Canada | EU082387 | EU082306 | - |
| Oxynaspis celata Darwin | Azores Islands | EU082412 | EU082331 | EU082371 |
| *Paralepas dannevigi* (Broch) | Australia | EU082399 | EU082318 | EU082358 |
| *Poecilasma inaequilaterale* Pilsbry | Gulf of Mexico | AY520654 | AY520620 | AY520722 |
| *Pollicipes polymerus* Sowerby | Monterey Bay, USA | AY520651 | AY520617 | AY520719 |
| *Scalpellum scalpellum* (Linnaeus) | Sweden | EU082388 | EU082307 | EU082347 |
| *Smilium peroni* (Gray) | Marmion, Australia | EU082386 | EU082305 | EU082346 |
| *Trianguloscalpellum regium* (Thomson) | Gulf of Mexico, USA | EU082389 | EU082308 | EU082348 |
| *Volcanolepas osheai* (Buckeridge) | Brothers Caldera, New Zealand | EU082394 | EU082313 | EU082353 |
| SESSILIA |  |  |  |  |
| Austromegabalanus psittacus (Molina) | Valdivia, Chile | AY520634 | AY520600 | AY520702 |
| Balanus balanus (Linnaeus) | Japan | AY520628 | AY520594 | AY520696 |
| *Catomerus polymerus* (Darwin) | Tasmania, Australia | AY520648 | AY520614 | AY520716 |
| *Chamaesipho tasmanica* Foster & Anderson | Tasmania, Australia | AY520647 | AY520613 | AY520715 |
| *Chelonibia patula* (Ranzani) | Israel | L26514 | EU082295 | EU082337 |
| *Chthamalus montagui* Southward | Vigo Bay, Spain | AY520642 | AY520608 | AY520710 |
| *Elminius kingii* Gray | Valdivia, Chile | AY520636 | AY520602 | AY520704 |
| *Jehlius cirratus* (Darwin) | Valdivia, Chile | AY520645 | AY520611 | AY520713 |
| *Megabalanus tintinnabulum* (Linnaeus) | Monterey Bay, USA | AY520631 | AY520597 | AY520699 |
| Menesiniella aquila (Pilsbry) | Monterey Bay, USA | AY520630 | AY520596 | AY520698 |
| *Metaverruca recta* (Aurivillius) | Ogasawara Islands, Japan | EU082378 | EU082297 | EU082339 |
| *Neoverruca brachylepadoformis* Newman & Hessler | Mariana Trough | EU082398 | EU082317 | EU082357 |
| *Notochthamalus scabrosus* Darwin | Valdivia, Chile | AY520646 | AY520612 | AY520714 |
| *Rostratoverruca krugeri* (Broch) | Nansei Islands, Japan | EU082380 | EU082299 | EU082341 |
| *Semibalanus cariosus* (Pallas) | Monterey Bay, USA | AY520627 | AY520593 | AY520695 |
| Tetraclita japonica Pilsbry | Japan | AY520640 | AY520606 | AY520708 |
| Tetraclitella divisa (Nilsson-Cantell) | Annobón, Equatorial Guinea | AY520637 | AY520603 | AY520705 |
| *Verruca stroemia* (Müller) | Vigo Bay, Spain | AY520649 | AY520615 | AY520717 |
| MALACOSTRACA |  |  |  |  |
| EUMALACOSTRACA |  |  |  |  |
| DECAPODA |  |  |  |  |
| *Homarus americanus* Milne-Edwards | GenBank | AF235971 | DQ079788 | DQ079675 |
| *Astacus astacus* (Linnaeus) | GenBank | AF235959 | DQ079773 | DQ079660 |
| *Cancer pagurus* Linnaeus | GenBank | DQ079743 | DQ079781 | DQ079668 |
| EUPHAUSIACEA |  |  |  |  |
| *Meganyctiphanes norvegica* (Sars) | GenBank | DQ900731 | AY744900 | AY744906 |
| STOMATOPODA |  |  |  |  |
| *Squilla empusa* Say | GenBank | L81946 | AY210842 | - |
| *Kempina mikado* (Kemp & Chopra) | GenBank | AF370802 | - | AF110873 |
| ISOPODA |  |  |  |  |
| *Asellus aquaticus* (Linnaeus) | GenBank | AF255701 | DQ144749 | AJ238321 |
| THERMOSBAENACEA |  |  |  |  |
| *Tethysbaena argentarii* (Stella) | GenBank | AY781415 | DQ470654 | - |
| CUMACEA |  |  |  |  |
| *Vaunthompsonia minor* Zimmer | GenBank | AY743938 | - | - |
| TANAIDACEA |  |  |  |  |
| *Paratanais malignus* Larsen | GenBank | AY781429 | - | - |
| AMPHIPODA |  |  |  |  |
| *Hyalella azteca* Saussure | GenBank | AY743944 | DQ464760 | - |
| PHYLLOCARIDA |  |  |  |  |
| LEPTOSTRACA |  |  |  |  |
| *Paranebalia longipes* (Willemoes-Suhm) | GenBank | AY744891 | EF189655 | AY744905 |
| COPEPODA |  |  |  |  |
| NEOCOPEPODA |  |  |  |  |
| GYMNOPLEA |  |  |  |  |
| *Neocalanus cristatus* (Krøyer) | GenBank | AF514344 | EF460776 | - |
| *Neocalanus plumchrus* (Murakawa) | GenBank | AF514340 | EF460778 | - |
| PODOPLEA |  |  |  |  |
| *Caligus elongatus* Nordmann | GenBank | AY627020 | DQ180337 | - |
| *Chondracanthus lophii* Johnston | GenBank | L34046 | DQ180341 | - |
| *Ergasilus anchoratus* Markewitsch | GenBank | DQ107564 | DQ107528 | - |
| *Lernaea cyprinacea* (Linnaeus) | GenBank | DQ107557 | DQ107548 | - |
| *Lamproglena orientalis* von Nordmann | GenBank | DQ107552 | DQ107544 | - |
| *Lepeophtheirus salmonis* (Krøyer) | GenBank | DQ123829 | DQ180342 | - |
| *Monstrilla clavata* Sars | GenBank | DQ538495 | - | - |
| *Tigriopus californicus* (Baker) | GenBank | AF363306 | AF363324 | - |

**Appendix 1.** Morphological characters and states used for the MP and BMCMC phylogenetic analyses. Those adapted from Grygier [8, 66] are also indicated by his characters numbers, but have been revised and recoded.

**01**: Naupliar cephalic shield continuous with free trunk dorsum [[66]: 01]

0 = no

1 = yes

**02**: Naupliar cephalic shield ridges of common (facetotectan) plan [[66]: 03]

0 = no

1 = yes

**03**: Nauplius with dorsal window plate in common position [[66]: 04]

0 = no

1 = yes

**04**: Nauplius with ventral side of cephalic region flat, round and with wide rim [[66]: 05]

0 = no

1 = yes

**05:** Nauplius with furcal setae; excluding furcal spines which apparently all taxa can have [[66]: 11]

0 = absent

1 = present

**06:** Frontolateral horns in nauplii; or the frontolateral pores in cyprids [[8]: 02]

0 = absent

1 = present

**07** (05) Frontal filaments in nauplius [[8]: 06]

0 = absent

1 = present

**08:** Antennular segment number expressed in entire life cycle [[8]: 04]

0 = 8 or more expressed

1 = less than 8 expressed

**09:** Naupliar antennular segments [[8]: 01]

0 = more than 3 segments in antennule of stage 1 nauplii

1 = 3 or fewer segments

**10:** Nauplii with at most two segments in endopods of antennae and mandibles [[8]: 02]

0 = no

1 = yes

**11:** Head shield with distinct hinge line

0 = absent

1 = present

**12:** Lattice organs

0 = absent

1 = present

Lattice organs are normally present in 5 pairs, but some taxa have, apparently secondarily, lost specific pairs. A more detailed coding would split character 12 into the presence of specific lattice organ pair, but the secondary loss of lattice organs seems only to be important at rather low taxonomic levels.

**13:** Lattice organs (Lo) shape

0 = with more or less distinct crest in trough

1 = no distinct crest

**14:** Lattice organ pair 1 (Lo1) terminal pore position

0 = posterior

1 = anterior

**15:** Lo2 terminal pore position

0 = posterior

1 = anterior

**16:** Lattice organs with pores (large or small)

0 = absent

1 = present

This concerns both the presence and small pores and large pores as dealt with in character 17

**17:** Lattice organs with deep pores (pits) almost reaching cuticular chamber [see 89]

0 = absent

1 = present

**18:** Well developed frontal filaments in cypridiform larva [[8]: 11]

0 = absent

1 = present

**19:** Basal part of cypridoid antennule consisting of a two articulating sclerites [= Y-rod and U-plate sensu Høeg [90]]

0 = absent

1 = present

This concerns the specialized morphology of first antennular segment in cirripede cyprids, which likely represents two segments [90, 91]

**20:** A hand, hoof or bell shaped semi-distal antennular segment (with disc or claw) in the cypridoid larva

0 = absent

1 = present

**21:** Cypridoid antennule with an attachment disc covered with cuticular villi

0 = absent

1 = present

**22:** Distal element of cypridoid antennule is a cylindrical, movable sensory “palp” (from two segments or two fused segments)

0 = absent

1 = present

**23:** Cypridoid antennular sensory palp undivided (segments fused)

0 = no

1 = yes

In cirripedes the “palp” in characters 22 and 23 is the 4^th^ antennular segment

**24:** Cypridoid antennule with (motile) distal claw for mechanical attachment

0 = absent

1 = present

We score “?” for facetotectans other than *Hansenocaris itoi*, because the y-cyprids of the undescribed species used in our analysis have not yet been studied for this feature

**25:** Aesthetascs

0 = on almost every antennular segment

1 = only one or two aesthetascs situated distally on the antennule

**26:** Reduction of distal antennular musculature in cypridoid larva [[8]: 14]

0= absent

1 = present

**27:** Multicellular cement gland exiting on cypridoid antennule

0 = absent

1 = present

**28:** Filamentary tuft-like cephalic sensory appendage present in post-naupliar stage [[8]: 12]

0 = absent

1 = present

**29:** Mouthparts and gut [[8]: 06]

0 = retained after metanauplius

1= lost or reduced in cypridoid stage, but can reappear

**30:** Postoral adductor muscle (for any post-naupliar stage)

0 = absent

1 = present

**31:** Reduction of thoracopodal musculature [[8]: 17]

0 = not reduced

1 = reduced

**32:** Two segmented endopods in post-naupliar thoracopods [[8]: 16]

0 = basal 2 segments not fused

1 = basal two segments fused

This refers to the fusion of the basal two segments of a three segmented endopod (excluding thoracopod 1 which can differ). We omit to code for the special character state seen in some Facetotecta, where fusion of the distal two segments leads to two segmented endopods. This is an ingroup facetotectan condition [8].

**33:** Seta on first exopodal segment in post-naupliar instars

0 = absent

1 = present

**34:** Single seta on post-naupliar first exopodal segment is a serrated grooming seta

0 = absent

1 = present

A seta is present in the Ascothoracida and Copepoda, but not as a distinctly serrated seta.

**35:** Abdominal development; excluding telson [8]

0 = well developed

1 = rudimentary or absent

**36:** Abdominal segment number, excluding telson [8]

0 = 4

1 = less than 4

We score similarly (1) for the Facetotecta and the Cirripedia. But facetotectans have a well developed abdomen while cirripedes have an abdomen that is either diminutive or indistinguishably fused with a small telson.

**37**: Telson-abdomen development relative to thorax [[8]: 19]

1 = not pronouncedly narrower than thorax and not set off

2= always distinct but narrower and set off from thorax

In state 2 acrothoracicans have an intervening abdomen isodiametrical with telson [states 35(1) and 36(0)].

**38:** Telson cleft

0 = no cleft

1 = distinct cleft, shallow to almost reaching the base

[State 2 has occasionally been misunderstood as a the presence of two segmented caudal rami, see Kolbasov and Høeg, [68]]

**39:** Profound metamorphosis after cypridoid stage

0 = absent

1 = present

**40:** Preoral adductor muscle in post-cypridoid stage

0 = absent

1 = present

**41:** Primordial shell plate in first attached instar

0 = no

1 = yes

|  |  |  |  |  |  |  |  |  |  |  |  |  |  |  |  |  |  |  |  |  |  |  |  |  |  |  |  |  |  |  |  |  |  |  |  |  |  |  |  |  |  |  |
| --- | --- | --- | --- | --- | --- | --- | --- | --- | --- | --- | --- | --- | --- | --- | --- | --- | --- | --- | --- | --- | --- | --- | --- | --- | --- | --- | --- | --- | --- | --- | --- | --- | --- | --- | --- | --- | --- | --- | --- | --- | --- | --- |
|  |  |  |  |  |  |  |  |  |  |  |  |  |  |  |  |  |  |  |  |  |  |  |  |  |  |  |  |  |  |  |  |  |  |  |  |  |  |  |  |  |  |  |
|  |  |  |  |  |  |  |  |  |  |  |  |  |  |  |  |  |  |  |  |  |  |  |  |  |  |  |  |  |  |  |  |  |  |  |  |  |  |  |  |  |  |  |
|  |  |  |  |  |  |  |  |  |  |  |  |  |  |  |  |  |  |  |  |  |  |  |  |  |  |  |  |  |  |  |  |  |  |  |  |  |  |  |  |  |  |  |
|  |  |  |  |  |  |  |  |  |  |  |  |  |  |  |  |  |  |  |  |  |  |  |  |  |  |  |  |  |  |  |  |  |  |  |  |  |  |  |  |  |  |  |
|  |  |  |  |  |  |  |  |  |  |  |  |  |  |  |  |  |  |  |  |  |  |  |  |  |  |  |  |  |  |  |  |  |  |  |  |  |  |  |  |  |  |  |
|  |  |  |  |  |  |  |  |  |  |  |  |  |  |  |  |  |  |  |  |  |  |  |  |  |  |  |  |  |  |  |  |  |  |  |  |  |  |  |  |  |  |  |
|  |  |  |  |  |  |  |  |  |  |  |  |  |  |  |  |  |  |  |  |  |  |  |  |  |  |  |  |  |  |  |  |  |  |  |  |  |  |  |  |  |  |  |
|  |  |  |  |  |  |  |  |  |  |  |  |  |  |  |  |  |  |  |  |  |  |  |  |  |  |  |  |  |  |  |  |  |  |  |  |  |  |  |  |  |  |  |
|  |  |  |  |  |  |  |  |  |  |  |  |  |  |  |  |  |  |  |  |  |  |  |  |  |  |  |  |  |  |  |  |  |  |  |  |  |  |  |  |  |  |  |
|  |  |  |  |  |  |  |  |  |  |  |  |  |  |  |  |  |  |  |  |  |  |  |  |  |  |  |  |  |  |  |  |  |  |  |  |  |  |  |  |  |  |  |
|  |  |  |  |  |  |  |  |  |  |  |  |  |  |  |  |  |  |  |  |  |  |  |  |  |  |  |  |  |  |  |  |  |  |  |  |  |  |  |  |  |  |  |
|  |  |  |  |  |  |  |  |  |  |  |  |  |  |  |  |  |  |  |  |  |  |  |  |  |  |  |  |  |  |  |  |  |  |  |  |  |  |  |  |  |  |  |
|  |  |  |  |  |  |  |  |  |  |  |  |  |  |  |  |  |  |  |  |  |  |  |  |  |  |  |  |  |  |  |  |  |  |  |  |  |  |  |  |  |  |  |
|  |  |  |  |  |  |  |  |  |  |  |  |  |  |  |  |  |  |  |  |  |  |  |  |  |  |  |  |  |  |  |  |  |  |  |  |  |  |  |  |  |  |  |
|  |  |  |  |  |  |  |  |  |  |  |  |  |  |  |  |  |  |  |  |  |  |  |  |  |  |  |  |  |  |  |  |  |  |  |  |  |  |  |  |  |  |  |
|  |  |  |  |  |  |  |  |  |  |  |  |  |  |  |  |  |  |  |  |  |  |  |  |  |  |  |  |  |  |  |  |  |  |  |  |  |  |  |  |  |  |  |
|  |  |  |  |  |  |  |  |  |  |  |  |  |  |  |  |  |  |  |  |  |  |  |  |  |  |  |  |  |  |  |  |  |  |  |  |  |  |  |  |  |  |  |
|  |  |  |  |  |  |  |  |  |  |  |  |  |  |  |  |  |  |  |  |  |  |  |  |  |  |  |  |  |  |  |  |  |  |  |  |  |  |  |  |  |  |  |
|  |  |  |  |  |  |  |  |  |  |  |  |  |  |  |  |  |  |  |  |  |  |  |  |  |  |  |  |  |  |  |  |  |  |  |  |  |  |  |  |  |  |  |
|  |  |  |  |  |  |  |  |  |  |  |  |  |  |  |  |  |  |  |  |  |  |  |  |  |  |  |  |  |  |  |  |  |  |  |  |  |  |  |  |  |  |  |
|  |  |  |  |  |  |  |  |  |  |  |  |  |  |  |  |  |  |  |  |  |  |  |  |  |  |  |  |  |  |  |  |  |  |  |  |  |  |  |  |  |  |  |
|  |  |  |  |  |  |  |  |  |  |  |  |  |  |  |  |  |  |  |  |  |  |  |  |  |  |  |  |  |  |  |  |  |  |  |  |  |  |  |  |  |  |  |
|  |  |  |  |  |  |  |  |  |  |  |  |  |  |  |  |  |  |  |  |  |  |  |  |  |  |  |  |  |  |  |  |  |  |  |  |  |  |  |  |  |  |  |
|  |  |  |  |  |  |  |  |  |  |  |  |  |  |  |  |  |  |  |  |  |  |  |  |  |  |  |  |  |  |  |  |  |  |  |  |  |  |  |  |  |  |  |
|  |  |  |  |  |  |  |  |  |  |  |  |  |  |  |  |  |  |  |  |  |  |  |  |  |  |  |  |  |  |  |  |  |  |  |  |  |  |  |  |  |  |  |
|  |  |  |  |  |  |  |  |  |  |  |  |  |  |  |  |  |  |  |  |  |  |  |  |  |  |  |  |  |  |  |  |  |  |  |  |  |  |  |  |  |  |  |
|  |  |  |  |  |  |  |  |  |  |  |  |  |  |  |  |  |  |  |  |  |  |  |  |  |  |  |  |  |  |  |  |  |  |  |  |  |  |  |  |  |  |  |
|  |  |  |  |  |  |  |  |  |  |  |  |  |  |  |  |  |  |  |  |  |  |  |  |  |  |  |  |  |  |  |  |  |  |  |  |  |  |  |  |  |  |  |
|  |  |  |  |  |  |  |  |  |  |  |  |  |  |  |  |  |  |  |  |  |  |  |  |  |  |  |  |  |  |  |  |  |  |  |  |  |  |  |  |  |  |  |
|  |  |  |  |  |  |  |  |  |  |  |  |  |  |  |  |  |  |  |  |  |  |  |  |  |  |  |  |  |  |  |  |  |  |  |  |  |  |  |  |  |  |  |
|  |  |  |  |  |  |  |  |  |  |  |  |  |  |  |  |  |  |  |  |  |  |  |  |  |  |  |  |  |  |  |  |  |  |  |  |  |  |  |  |  |  |  |
|  |  |  |  |  |  |  |  |  |  |  |  |  |  |  |  |  |  |  |  |  |  |  |  |  |  |  |  |  |  |  |  |  |  |  |  |  |  |  |  |  |  |  |
|  |  |  |  |  |  |  |  |  |  |  |  |  |  |  |  |  |  |  |  |  |  |  |  |  |  |  |  |  |  |  |  |  |  |  |  |  |  |  |  |  |  |  |
|  |  |  |  |  |  |  |  |  |  |  |  |  |  |  |  |  |  |  |  |  |  |  |  |  |  |  |  |  |  |  |  |  |  |  |  |  |  |  |  |  |  |  |
|  |  |  |  |  |  |  |  |  |  |  |  |  |  |  |  |  |  |  |  |  |  |  |  |  |  |  |  |  |  |  |  |  |  |  |  |  |  |  |  |  |  |  |
|  |  |  |  |  |  |  |  |  |  |  |  |  |  |  |  |  |  |  |  |  |  |  |  |  |  |  |  |  |  |  |  |  |  |  |  |  |  |  |  |  |  |  |
|  |  |  |  |  |  |  |  |  |  |  |  |  |  |  |  |  |  |  |  |  |  |  |  |  |  |  |  |  |  |  |  |  |  |  |  |  |  |  |  |  |  |  |
|  |  |  |  |  |  |  |  |  |  |  |  |  |  |  |  |  |  |  |  |  |  |  |  |  |  |  |  |  |  |  |  |  |  |  |  |  |  |  |  |  |  |  |
|  |  |  |  |  |  |  |  |  |  |  |  |  |  |  |  |  |  |  |  |  |  |  |  |  |  |  |  |  |  |  |  |  |  |  |  |  |  |  |  |  |  |  |
|  |  |  |  |  |  |  |  |  |  |  |  |  |  |  |  |  |  |  |  |  |  |  |  |  |  |  |  |  |  |  |  |  |  |  |  |  |  |  |  |  |  |  |
|  |  |  |  |  |  |  |  |  |  |  |  |  |  |  |  |  |  |  |  |  |  |  |  |  |  |  |  |  |  |  |  |  |  |  |  |  |  |  |  |  |  |  |
|  |  |  |  |  |  |  |  |  |  |  |  |  |  |  |  |  |  |  |  |  |  |  |  |  |  |  |  |  |  |  |  |  |  |  |  |  |  |  |  |  |  |  |
|  |  |  |  |  |  |  |  |  |  |  |  |  |  |  |  |  |  |  |  |  |  |  |  |  |  |  |  |  |  |  |  |  |  |  |  |  |  |  |  |  |  |  |
|  |  |  |  |  |  |  |  |  |  |  |  |  |  |  |  |  |  |  |  |  |  |  |  |  |  |  |  |  |  |  |  |  |  |  |  |  |  |  |  |  |  |  |
|  |  |  |  |  |  |  |  |  |  |  |  |  |  |  |  |  |  |  |  |  |  |  |  |  |  |  |  |  |  |  |  |  |  |  |  |  |  |  |  |  |  |  |
|  |  |  |  |  |  |  |  |  |  |  |  |  |  |  |  |  |  |  |  |  |  |  |  |  |  |  |  |  |  |  |  |  |  |  |  |  |  |  |  |  |  |  |
|  |  |  |  |  |  |  |  |  |  |  |  |  |  |  |  |  |  |  |  |  |  |  |  |  |  |  |  |  |  |  |  |  |  |  |  |  |  |  |  |  |  |  |
|  |  |  |  |  |  |  |  |  |  |  |  |  |  |  |  |  |  |  |  |  |  |  |  |  |  |  |  |  |  |  |  |  |  |  |  |  |  |  |  |  |  |  |
|  |  |  |  |  |  |  |  |  |  |  |  |  |  |  |  |  |  |  |  |  |  |  |  |  |  |  |  |  |  |  |  |  |  |  |  |  |  |  |  |  |  |  |
|  |  |  |  |  |  |  |  |  |  |  |  |  |  |  |  |  |  |  |  |  |  |  |  |  |  |  |  |  |  |  |  |  |  |  |  |  |  |  |  |  |  |  |
|  |  |  |  |  |  |  |  |  |  |  |  |  |  |  |  |  |  |  |  |  |  |  |  |  |  |  |  |  |  |  |  |  |  |  |  |  |  |  |  |  |  |  |
|  |  |  |  |  |  |  |  |  |  |  |  |  |  |  |  |  |  |  |  |  |  |  |  |  |  |  |  |  |  |  |  |  |  |  |  |  |  |  |  |  |  |  |
|  |  |  |  |  |  |  |  |  |  |  |  |  |  |  |  |  |  |  |  |  |  |  |  |  |  |  |  |  |  |  |  |  |  |  |  |  |  |  |  |  |  |  |
|  |  |  |  |  |  |  |  |  |  |  |  |  |  |  |  |  |  |  |  |  |  |  |  |  |  |  |  |  |  |  |  |  |  |  |  |  |  |  |  |  |  |  |
|  |  |  |  |  |  |  |  |  |  |  |  |  |  |  |  |  |  |  |  |  |  |  |  |  |  |  |  |  |  |  |  |  |  |  |  |  |  |  |  |  |  |  |
|  |  |  |  |  |  |  |  |  |  |  |  |  |  |  |  |  |  |  |  |  |  |  |  |  |  |  |  |  |  |  |  |  |  |  |  |  |  |  |  |  |  |  |
|  |  |  |  |  |  |  |  |  |  |  |  |  |  |  |  |  |  |  |  |  |  |  |  |  |  |  |  |  |  |  |  |  |  |  |  |  |  |  |  |  |  |  |
|  |  |  |  |  |  |  |  |  |  |  |  |  |  |  |  |  |  |  |  |  |  |  |  |  |  |  |  |  |  |  |  |  |  |  |  |  |  |  |  |  |  |  |
|  |  |  |  |  |  |  |  |  |  |  |  |  |  |  |  |  |  |  |  |  |  |  |  |  |  |  |  |  |  |  |  |  |  |  |  |  |  |  |  |  |  |  |
|  |  |  |  |  |  |  |  |  |  |  |  |  |  |  |  |  |  |  |  |  |  |  |  |  |  |  |  |  |  |  |  |  |  |  |  |  |  |  |  |  |  |  |
|  |  |  |  |  |  |  |  |  |  |  |  |  |  |  |  |  |  |  |  |  |  |  |  |  |  |  |  |  |  |  |  |  |  |  |  |  |  |  |  |  |  |  |
|  |  |  |  |  |  |  |  |  |  |  |  |  |  |  |  |  |  |  |  |  |  |  |  |  |  |  |  |  |  |  |  |  |  |  |  |  |  |  |  |  |  |  |
|  |  |  |  |  |  |  |  |  |  |  |  |  |  |  |  |  |  |  |  |  |  |  |  |  |  |  |  |  |  |  |  |  |  |  |  |  |  |  |  |  |  |  |
|  |  |  |  |  |  |  |  |  |  |  |  |  |  |  |  |  |  |  |  |  |  |  |  |  |  |  |  |  |  |  |  |  |  |  |  |  |  |  |  |  |  |  |
|  |  |  |  |  |  |  |  |  |  |  |  |  |  |  |  |  |  |  |  |  |  |  |  |  |  |  |  |  |  |  |  |  |  |  |  |  |  |  |  |  |  |  |
|  |  |  |  |  |  |  |  |  |  |  |  |  |  |  |  |  |  |  |  |  |  |  |  |  |  |  |  |  |  |  |  |  |  |  |  |  |  |  |  |  |  |  |
|  |  |  |  |  |  |  |  |  |  |  |  |  |  |  |  |  |  |  |  |  |  |  |  |  |  |  |  |  |  |  |  |  |  |  |  |  |  |  |  |  |  |  |
|  |  |  |  |  |  |  |  |  |  |  |  |  |  |  |  |  |  |  |  |  |  |  |  |  |  |  |  |  |  |  |  |  |  |  |  |  |  |  |  |  |  |  |
|  |  |  |  |  |  |  |  |  |  |  |  |  |  |  |  |  |  |  |  |  |  |  |  |  |  |  |  |  |  |  |  |  |  |  |  |  |  |  |  |  |  |  |
|  |  |  |  |  |  |  |  |  |  |  |  |  |  |  |  |  |  |  |  |  |  |  |  |  |  |  |  |  |  |  |  |  |  |  |  |  |  |  |  |  |  |  |
|  |  |  |  |  |  |  |  |  |  |  |  |  |  |  |  |  |  |  |  |  |  |  |  |  |  |  |  |  |  |  |  |  |  |  |  |  |  |  |  |  |  |  |
|  |  |  |  |  |  |  |  |  |  |  |  |  |  |  |  |  |  |  |  |  |  |  |  |  |  |  |  |  |  |  |  |  |  |  |  |  |  |  |  |  |  |  |
|  |  |  |  |  |  |  |  |  |  |  |  |  |  |  |  |  |  |  |  |  |  |  |  |  |  |  |  |  |  |  |  |  |  |  |  |  |  |  |  |  |  |  |
|  |  |  |  |  |  |  |  |  |  |  |  |  |  |  |  |  |  |  |  |  |  |  |  |  |  |  |  |  |  |  |  |  |  |  |  |  |  |  |  |  |  |  |
|  |  |  |  |  |  |  |  |  |  |  |  |  |  |  |  |  |  |  |  |  |  |  |  |  |  |  |  |  |  |  |  |  |  |  |  |  |  |  |  |  |  |  |
|  |  |  |  |  |  |  |  |  |  |  |  |  |  |  |  |  |  |  |  |  |  |  |  |  |  |  |  |  |  |  |  |  |  |  |  |  |  |  |  |  |  |  |
|  |  |  |  |  |  |  |  |  |  |  |  |  |  |  |  |  |  |  |  |  |  |  |  |  |  |  |  |  |  |  |  |  |  |  |  |  |  |  |  |  |  |  |
|  |  |  |  |  |  |  |  |  |  |  |  |  |  |  |  |  |  |  |  |  |  |  |  |  |  |  |  |  |  |  |  |  |  |  |  |  |  |  |  |  |  |  |
|  |  |  |  |  |  |  |  |  |  |  |  |  |  |  |  |  |  |  |  |  |  |  |  |  |  |  |  |  |  |  |  |  |  |  |  |  |  |  |  |  |  |  |
|  |  |  |  |  |  |  |  |  |  |  |  |  |  |  |  |  |  |  |  |  |  |  |  |  |  |  |  |  |  |  |  |  |  |  |  |  |  |  |  |  |  |  |
|  |  |  |  |  |  |  |  |  |  |  |  |  |  |  |  |  |  |  |  |  |  |  |  |  |  |  |  |  |  |  |  |  |  |  |  |  |  |  |  |  |  |  |
|  |  |  |  |  |  |  |  |  |  |  |  |  |  |  |  |  |  |  |  |  |  |  |  |  |  |  |  |  |  |  |  |  |  |  |  |  |  |  |  |  |  |  |
|  |  |  |  |  |  |  |  |  |  |  |  |  |  |  |  |  |  |  |  |  |  |  |  |  |  |  |  |  |  |  |  |  |  |  |  |  |  |  |  |  |  |  |
|  |  |  |  |  |  |  |  |  |  |  |  |  |  |  |  |  |  |  |  |  |  |  |  |  |  |  |  |  |  |  |  |  |  |  |  |  |  |  |  |  |  |  |
|  |  |  |  |  |  |  |  |  |  |  |  |  |  |  |  |  |  |  |  |  |  |  |  |  |  |  |  |  |  |  |  |  |  |  |  |  |  |  |  |  |  |  |
|  |  |  |  |  |  |  |  |  |  |  |  |  |  |  |  |  |  |  |  |  |  |  |  |  |  |  |  |  |  |  |  |  |  |  |  |  |  |  |  |  |  |  |
|  |  |  |  |  |  |  |  |  |  |  |  |  |  |  |  |  |  |  |  |  |  |  |  |  |  |  |  |  |  |  |  |  |  |  |  |  |  |  |  |  |  |  |
|  |  |  |  |  |  |  |  |  |  |  |  |  |  |  |  |  |  |  |  |  |  |  |  |  |  |  |  |  |  |  |  |  |  |  |  |  |  |  |  |  |  |  |
|  |  |  |  |  |  |  |  |  |  |  |  |  |  |  |  |  |  |  |  |  |  |  |  |  |  |  |  |  |  |  |  |  |  |  |  |  |  |  |  |  |  |  |
|  |  |  |  |  |  |  |  |  |  |  |  |  |  |  |  |  |  |  |  |  |  |  |  |  |  |  |  |  |  |  |  |  |  |  |  |  |  |  |  |  |  |  |
|  |  |  |  |  |  |  |  |  |  |  |  |  |  |  |  |  |  |  |  |  |  |  |  |  |  |  |  |  |  |  |  |  |  |  |  |  |  |  |  |  |  |  |
|  |  |  |  |  |  |  |  |  |  |  |  |  |  |  |  |  |  |  |  |  |  |  |  |  |  |  |  |  |  |  |  |  |  |  |  |  |  |  |  |  |  |  |

| Naup/Cyp/Metamorph | N | N | N | N | N | N | N | N | N | N | C | C | C | C | C | C | C | C | C | C | C | C | C | C | C | C | C | C | C | C | C | C | C | C | C | C | C | C | M | M | M |
| --- | --- | --- | --- | --- | --- | --- | --- | --- | --- | --- | --- | --- | --- | --- | --- | --- | --- | --- | --- | --- | --- | --- | --- | --- | --- | --- | --- | --- | --- | --- | --- | --- | --- | --- | --- | --- | --- | --- | --- | --- | --- |
| Character # | 1 | 2 | 3 | 4 | 5 | 6 | 7 | 8 | 9 | 10 | 11 | 12 | 13 | 14 | 15 | 16 | 17 | 18 | 19 | 20 | 21 | 22 | 23 | 24 | 25 | 26 | 27 | 28 | 29 | 30 | 31 | 32 | 33 | 34 | 35 | 36 | 37 | 38 | 39 | 40 | 41 |
| Grygier (1987) character # |  |  |  |  |  |  |  | 4 | 1 | 2 |  |  |  |  |  |  |  |  |  |  |  |  |  |  |  | 14 |  | 12 | 6 |  | 17 | 16 |  |  | 19 | 19 | 19 |  |  |  |  |
| Grygier (1991) character # |  |  |  |  |  |  | 6 |  |  |  |  |  |  |  |  |  |  |  |  |  |  |  |  |  |  |  |  |  |  |  |  |  |  |  |  |  |  |  |  |  |  |
| *Neocalanus plumchrus* | 0 | 0 | 0 | 0 | 1 | 0 | 0 | 0 | 1 | 1 | 0 | 0 | ? | ? | ? | ? | ? | 0 | 0 | ? | ? | 0 | ? | ? | 0 | 0 | 0 | 0 | 0 | 0 | 0 | 0 | 1 | 0 | 0 | 0 | 0 | 0 | ? | 0 | ? |
| *Neocalanus cristatus* | 0 | 0 | 0 | 0 | 1 | 0 | 0 | 0 | 1 | 1 | 0 | 0 | ? | ? | ? | ? | ? | 0 | 0 | ? | ? | 0 | ? | ? | 0 | 0 | 0 | 0 | 0 | 0 | 0 | 0 | 1 | 0 | 0 | 0 | 0 | 0 | ? | 0 | ? |
| *Tigriopus californicus* | 0 | 0 | 0 | 0 | 1 | 0 | 0 | 0 | 1 | 1 | 0 | 0 | ? | ? | ? | ? | ? | 0 | 0 | ? | ? | 0 | ? | ? | 0 | 0 | 0 | 0 | 0 | 0 | 0 | 0 | 1 | 0 | 0 | 0 | 0 | 0 | ? | 0 | ? |
| *Monstrilla clavata* | 0 | 0 | 0 | 0 | 1 | 0 | 0 | 0 | 1 | 1 | 0 | 0 | ? | ? | ? | ? | ? | 0 | 0 | ? | ? | 0 | ? | ? | 0 | 0 | 0 | 0 | ? | 0 | 0 | 0 | 1 | 0 | 0 | 0 | 0 | 0 | ? | 0 | ? |
| *Lepeoptheirus salmonensis* | 0 | 0 | 0 | 0 | 1 | 0 | 0 | 0 | 1 | 1 | 0 | 0 | ? | ? | ? | ? | ? | 0 | 0 | ? | ? | 0 | ? | ? | 0 | 0 | 0 | 0 | 0 | 0 | 0 | 0 | 1 | 0 | 0 | 0 | 0 | 0 | ? | 0 | ? |
| *Caligus elongatus* | 0 | 0 | 0 | 0 | 1 | 0 | 0 | 0 | 1 | 1 | 0 | 0 | ? | ? | ? | ? | ? | 0 | 0 | ? | ? | 0 | ? | ? | 0 | 0 | 0 | 0 | 0 | 0 | 0 | 0 | 1 | 0 | 0 | 0 | 0 | 0 | ? | 0 | ? |
| *Chondracanthus lophii* | 0 | 0 | 0 | 0 | 1 | 0 | 0 | 0 | 1 | 1 | 0 | 0 | ? | ? | ? | ? | ? | 0 | 0 | ? | ? | 0 | ? | ? | 0 | 0 | 0 | 0 | ? | 0 | 0 | 0 | 1 | 0 | 0 | 0 | 0 | 0 | ? | 0 | ? |
| *Ergasilus anchoratus* | 0 | 0 | 0 | 0 | 1 | 0 | 0 | 0 | 1 | 1 | 0 | 0 | ? | ? | ? | ? | ? | 0 | 0 | ? | ? | 0 | ? | ? | 0 | 0 | 0 | 0 | 0 | 0 | 0 | 0 | 1 | 0 | 0 | 0 | 0 | 0 | ? | 0 | ? |
| *Lernaea cyprinacea* | 0 | 0 | 0 | 0 | 1 | 0 | 0 | 0 | 1 | 1 | 0 | 0 | ? | ? | ? | ? | ? | 0 | 0 | ? | ? | 0 | ? | ? | 0 | 0 | 0 | 0 | ? | 0 | 0 | 0 | 1 | 0 | 0 | 0 | 0 | 0 | ? | 0 | ? |
| *Laproglena orientalis* | 0 | 0 | 0 | 0 | 1 | 0 | 0 | 0 | 1 | 1 | 0 | 0 | ? | ? | ? | ? | ? | 0 | 0 | ? | ? | 0 | ? | ? | 0 | 0 | 0 | 0 | ? | 0 | 0 | 0 | 1 | 0 | 0 | 0 | 0 | 0 | ? | 0 | ? |
| *Homarus americanus* | ? | ? | ? | ? | ? | ? | ? | 1 | ? | ? | 0 | 0 | ? | ? | ? | ? | ? | ? | ? | ? | ? | ? | ? | ? | 0 | ? | ? | 0 | 0 | 0 | 0 | ? | ? | ? | 0 | ? | 0 | 0 | ? | 0 | ? |
| *Astacus astacus* | ? | ? | ? | ? | ? | ? | ? | 1 | ? | ? | 0 | 0 | ? | ? | ? | ? | ? | ? | ? | ? | ? | ? | ? | ? | 0 | ? | ? | 0 | 0 | 0 | 0 | ? | ? | ? | 0 | ? | 0 | 0 | ? | 0 | ? |
| *Cancer pagurus* | ? | ? | ? | ? | ? | ? | ? | 1 | ? | ? | 0 | 0 | ? | ? | ? | ? | ? | ? | ? | ? | ? | ? | ? | ? | 0 | ? | ? | 0 | 0 | 0 | 0 | ? | ? | ? | 0 | ? | 0 | 0 | ? | 0 | ? |
| *Squilla empusa* | ? | ? | ? | ? | ? | ? | ? | 1 | ? | ? | 0 | 0 | ? | ? | ? | ? | ? | ? | ? | ? | ? | ? | ? | ? | 0 | ? | ? | 0 | 0 | 0 | 0 | ? | ? | ? | 0 | ? | 0 | 0 | ? | 0 | ? |
| *Paranebalia longipes* | ? | ? | ? | ? | ? | ? | ? | 1 | ? | ? | 0 | 0 | ? | ? | ? | ? | ? | ? | ? | ? | ? | ? | ? | ? | 0 | ? | ? | 0 | 0 | 1 | 0 | ? | ? | ? | 0 | ? | 0 | 0 | ? | 0 | ? |
| *Meganyctiphanes norvegica* | ? | ? | ? | ? | ? | ? | ? | 1 | ? | ? | 0 | 0 | ? | ? | ? | ? | ? | ? | ? | ? | ? | ? | ? | ? | 0 | ? | ? | 0 | 0 | 0 | 0 | ? | ? | ? | 0 | ? | 0 | 0 | ? | 0 | ? |
| *Kempina mikado* | ? | ? | ? | ? | ? | ? | ? | 1 | ? | ? | 0 | 0 | ? | ? | ? | ? | ? | ? | ? | ? | ? | ? | ? | ? | 0 | ? | ? | 0 | 0 | 0 | 0 | ? | ? | ? | 0 | ? | 0 | 0 | ? | 0 | ? |
| *Asellus aquaticus* | ? | ? | ? | ? | ? | ? | ? | 1 | ? | ? | 0 | 0 | ? | ? | ? | ? | ? | ? | ? | ? | ? | ? | ? | ? | 0 | ? | ? | 0 | 0 | 0 | 0 | ? | ? | ? | 0 | ? | 0 | 0 | ? | 0 | ? |
| *Tethysbaena argentarii* | ? | ? | ? | ? | ? | ? | ? | 1 | ? | ? | 0 | 0 | ? | ? | ? | ? | ? | ? | ? | ? | ? | ? | ? | ? | 0 | ? | ? | 0 | 0 | 0 | 0 | ? | ? | ? | 0 | ? | 0 | 0 | ? | 0 | ? |
| *Vaunthompsonia minor* | ? | ? | ? | ? | ? | ? | ? | 1 | ? | ? | 0 | 0 | ? | ? | ? | ? | ? | ? | ? | ? | ? | ? | ? | ? | 0 | ? | ? | 0 | 0 | 0 | 0 | ? | ? | ? | 0 | ? | 0 | 0 | ? | 0 | ? |
| *Paratanais malignus* | ? | ? | ? | ? | ? | ? | ? | 1 | ? | ? | 0 | 0 | ? | ? | ? | ? | ? | ? | ? | ? | ? | ? | ? | ? | 0 | ? | ? | 0 | 0 | 0 | 0 | ? | ? | ? | 0 | ? | 0 | 0 | ? | 0 | ? |
| *Hyalella azteca* | ? | ? | ? | ? | ? | ? | ? | 1 | ? | ? | 0 | 0 | ? | ? | ? | ? | ? | ? | ? | ? | ? | ? | ? | ? | 0 | ? | ? | 0 | 0 | 0 | 0 | ? | ? | ? | 0 | ? | 0 | 0 | ? | 0 | ? |
| Facetotecta sp1 | 1 | 1 | 1 | 1 | 0 | 0 | 0 | 1 | 1 | 1 | 0 | ? | ? | ? | ? | ? | ? | 1 | 0 | 1 | 0 | 1 | 0 | ? | 1 | 0 | 0 | 1 | 1 | 0 | 1 | 0 | 0 | ? | 0 | 1 | 0 | 0 | 1 | 0 | 0 |
| Facetotecta sp2 | 1 | 1 | 1 | 1 | 0 | 0 | 0 | 1 | 1 | 1 | 0 | ? | ? | ? | ? | ? | ? | 1 | 0 | 1 | 0 | 1 | 0 | ? | 1 | 0 | 0 | 1 | 1 | 0 | 1 | 0 | 0 | ? | 0 | 1 | 0 | 0 | 1 | 0 | 0 |
| Facetotecta sp3 | 1 | 1 | 1 | 1 | 0 | 0 | 0 | 1 | 1 | 1 | 0 | ? | ? | ? | ? | ? | ? | 1 | 0 | 1 | 0 | 1 | 0 | ? | 1 | 0 | 0 | 1 | 1 | 0 | 1 | 0 | 0 | ? | 0 | 1 | 0 | 0 | 1 | 0 | 0 |
| Facetotecta sp4 | 1 | 1 | 1 | 1 | 0 | 0 | 0 | 1 | 1 | 1 | 0 | ? | ? | ? | ? | ? | ? | 1 | 0 | 1 | 0 | 1 | 0 | ? | 1 | 0 | 0 | 1 | 1 | 0 | 1 | 0 | 0 | ? | 0 | 1 | 0 | 0 | 1 | 0 | 0 |
| Facetotecta sp5 | 1 | 1 | 1 | 1 | 0 | 0 | 0 | 1 | 1 | 1 | 0 | ? | ? | ? | ? | ? | ? | 1 | 0 | 1 | 0 | 1 | 0 | ? | 1 | 0 | 0 | 1 | 1 | 0 | 1 | 0 | 0 | ? | 0 | 1 | 0 | 0 | 1 | 0 | 0 |
| Facetotecta sp6 | 1 | 1 | 1 | 1 | 0 | 0 | 0 | 1 | 1 | 1 | 0 | ? | ? | ? | ? | ? | ? | 1 | 0 | 1 | 0 | 1 | 0 | ? | 1 | 0 | 0 | 1 | 1 | 0 | 1 | 0 | 0 | ? | 0 | 1 | 0 | 0 | 1 | 0 | 0 |
| *Hansenocaris itoi* | 1 | 1 | 1 | 1 | 0 | 0 | 0 | 1 | 1 | 1 | 0 | 1 | 0 | 0 | 0 | 0 | 0 | 1 | 0 | 1 | 0 | 1 | 0 | 1 | 1 | 0 | 0 | 1 | 1 | 0 | 1 | 0 | 0 | ? | 0 | 1 | 0 | 0 | 1 | 0 | 0 |
| *Ulophysema oeresundense* | 0 | 0 | 0 | 0 | 1 | 0 | ? | 0 | ? | 0 | 1 | 1 | 0 | 0 | 0 | 0 | 0 | 1 | 0 | 0 | 0 | 0 | ? | 1 | 1 | 1 | 0 | 1 | 0 | 1 | 0 | 0 | 1 | 0 | 0 | ? | 0 | 0 | 0 | 0 | 0 |
| *Zibrowia auriculata* | 0 | 0 | 0 | 0 | 1 | 0 | 1 | 0 | 0 | 0 | 1 | 1 | ? | ? | ? | ? | ? | 1 | 0 | 0 | 0 | 0 | ? | 1 | 1 | 1 | 0 | 1 | 0 | 1 | 0 | 0 | 1 | 0 | 0 | 0 | 0 | 0 | 0 | 0 | 0 |
| *Baccalaureus maldiviensis* | 0 | ? | 0 | 0 | 1 | 0 | 1 | 0 | 0 | 0 | 1 | 1 | ? | ? | ? | ? | ? | 1 | 0 | 0 | 0 | 0 | ? | 1 | 1 | 1 | 0 | 1 | 0 | 1 | 0 | 0 | 1 | 0 | 0 | 0 | 0 | 0 | 0 | 0 | 0 |
| *Dendrogaster asterinae* | ? | ? | ? | ? | ? | 0 | ? | 0 | ? | ? | 1 | 1 | 0 | 0 | 0 | 0 | 0 | 1 | 0 | 0 | 0 | 0 | ? | 1 | 1 | 1 | 0 | 1 | 0 | 1 | 0 | 0 | 1 | 0 | 0 | ? | 0 | 0 | 0 | 0 | 0 |
| *Dendrogaster ludwigii* | ? | ? | ? | ? | ? | 0 | ? | 0 | ? | ? | 1 | 1 | 0 | 0 | 0 | 0 | 0 | 1 | 0 | 0 | 0 | 0 | ? | 1 | 1 | 1 | 0 | 1 | 0 | 1 | 0 | 0 | 1 | 0 | 0 | ? | 0 | 0 | 0 | 0 | 0 |
| *Trypetesa lampas* | 0 | 0 | 0 | 0 | 0 | 1 | 1 | 1 | 1 | 1 | 0 | 1 | 0 | 0 | 1 | 1 | 0 | 1 | 1 | 1 | 1 | 1 | 1 | 0 | 1 | 1 | 1 | 0 | 1 | 1 | 1 | 0 | 1 | 1 | 1 | 0 | 1 | 1 | 1 | 0 | 0 |
| *Auritoglyptes bicornis* | ? | ? | ? | ? | ? | ? | ? | ? | ? | ? | 0 | 1 | 0 | 0 | 1 | 1 | 0 | ? | 1 | 1 | 1 | 1 | 1 | 0 | 1 | 1 | 1 | 0 | 1 | 1 | 1 | 0 | 1 | 1 | 1 | 0 | 1 | 1 | 1 | 0 | 0 |
| *Berndtia purpurea* | ? | ? | ? | ? | ? | 1 | ? | ? | ? | ? | 0 | 1 | 0 | ? | ? | ? | ? | ? | 1 | 1 | 1 | 1 | 1 | 0 | ? | 1 | 1 | 0 | 1 | 1 | 1 | 0 | 1 | 1 | 1 | 0 | 1 | 1 | 1 | 0 | 0 |
| *Peltogaster paguri* | 0 | 0 | 0 | 0 | 0 | 1 | 1 | 1 | 1 | 1 | 0 | 1 | 1 | 1 | 1 | 1 | 1 | 1 | 1 | 1 | 1 | 1 | 1 | 0 | 1 | 1 | 1 | 0 | 1 | 1 | 1 | ? | 1 | 1 | 1 | 1 | 1 | 1 | 1 | ? | 0 |
| *Septosaccus rodriguezii* | 0 | 0 | 0 | 0 | 0 | 1 | 1 | 1 | 1 | 1 | 0 | ? | ? | ? | ? | ? | ? | 1 | 1 | 1 | 1 | 1 | 1 | 0 | 1 | 1 | 1 | 0 | 1 | 1 | 1 | ? | 1 | 1 | 1 | 1 | ? | ? | 1 | ? | ? |
| *Peltogasterella sulcata* | 0 | 0 | 0 | 0 | 0 | 1 | 1 | 1 | 1 | 1 | 0 | 1 | 1 | 1 | 1 | 1 | 1 | 1 | 1 | 1 | 1 | 1 | 1 | 0 | 1 | 1 | 1 | 0 | 1 | 1 | 1 | 1 | 1 | 1 | 1 | 1 | 1 | ? | 1 | ? | 0 |
| *Lernaeodiscus porcellanae* | 0 | 0 | 0 | 0 | 0 | 1 | 1 | 1 | 1 | 1 | 0 | 1 | 1 | 1 | 1 | 1 | 1 | 1 | 1 | 1 | 1 | 1 | 1 | 0 | 1 | 1 | 1 | 0 | 1 | 1 | 1 | 1 | 1 | 1 | 1 | 1 | 1 | ? | 1 | ? | 0 |
| *Sacculina carcini* | 0 | 0 | 0 | 0 | 0 | 1 | 1 | 1 | 1 | 1 | 0 | 1 | 1 | 1 | 1 | 1 | 1 | 1 | 1 | 1 | 1 | 1 | 1 | 0 | 1 | 1 | 1 | 0 | 1 | 1 | 1 | 1 | 1 | 1 | 1 | 1 | 1 | ? | 1 | ? | 0 |
| *Heterosaccus californicus* | 0 | 0 | 0 | 0 | 0 | 1 | 1 | 1 | 1 | 1 | 0 | 1 | 1 | 1 | 1 | 1 | 1 | 1 | 1 | 1 | 1 | 1 | 1 | 0 | 1 | 1 | 1 | 0 | 1 | 1 | 1 | 1 | 1 | 1 | 1 | 1 | 1 | ? | 1 | ? | ? |
| *Heterosaccus dollfusi* | 0 | ? | ? | ? | ? | 1 | 1 | ? | 1 | 1 | ? | ? | ? | ? | ? | ? | ? | 1 | 1 | 1 | 1 | 1 | 1 | 0 | ? | 1 | 1 | ? | 1 | 1 | 1 | ? | ? | ? | 1 | 1 | ? | ? | 1 | ? | ? |
| *Heterosaccus lunatus* | ? | 0 | 0 | 0 | 0 | 1 | 1 | 1 | 1 | 1 | 0 | 1 | 1 | 1 | 1 | 1 | 1 | 1 | 1 | 1 | 1 | 1 | 1 | 0 | 1 | 1 | 1 | 0 | 1 | 1 | 1 | ? | ? | ? | 1 | 1 | 1 | ? | 1 | ? | ? |
| *Loxothylacus texanus* | 0 | 0 | 0 | 0 | 0 | ? | ? | 1 | ? | ? | 0 | ? | ? | ? | ? | ? | ? | 1 | 1 | 1 | 1 | 1 | 1 | 0 | 1 | 1 | 1 | 0 | 1 | 1 | 1 | ? | ? | ? | 1 | 1 | ? | ? | 1 | ? | 0 |
| *Loxothylacus panopaei* | 0 | 0 | 0 | 0 | 0 | 1 | ? | 1 | ? | ? | 0 | 1 | 1 | 1 | 1 | 1 | 1 | 1 | 1 | 1 | 1 | 1 | 1 | 0 | 1 | 1 | 1 | 0 | 1 | 1 | 1 | ? | ? | ? | 1 | 1 | 1 | ? | 1 | ? | 0 |
| *Parthenopea subterranea* | 0 | 0 | 0 | 0 | 0 | 1 | 0 | ? | 1 | 1 | ? | ? | ? | ? | ? | ? | ? | ? | 1 | 1 | 1 | ? | ? | 0 | ? | 1 | 1 | ? | 1 | 1 | 1 | ? | ? | ? | ? | 1 | ? | ? | 1 | ? | ? |
| *Boschmaella japonica* | ? | ? | ? | ? | ? | ? | ? | 1 | ? | ? | 0 | ? | ? | ? | ? | ? | ? | ? | 1 | 1 | 1 | 0 | ? | ? | ? | 1 | 1 | 0 | 1 | 1 | 1 | ? | ? | ? | 1 | ? | 1 | ? | 1 | ? | ? |
| *Polysaccus japonicus* | ? | ? | ? | ? | ? | ? | ? | 1 | ? | ? | 0 | ? | ? | ? | ? | ? | ? | ? | 1 | ? | 1 | 1 | 1 | 0 | ? | 1 | 1 | 0 | 1 | 1 | 1 | ? | ? | ? | 1 | ? | 1 | ? | 1 | ? | ? |
| *Sylon hippolytes* | ? | ? | ? | ? | ? | 0 | ? | 1 | ? | ? | 0 | 1 | 1 | ? | ? | 1 | 1 | 0 | 1 | 1 | 1 | 1 | 1 | 0 | ? | 1 | 1 | 0 | 1 | 1 | 1 | 1 | 1 | 1 | 1 | 1 | 1 | 1 | 1 | ? | 0 |
| *Pottsia serenei* | ? | ? | ? | ? | ? | ? | ? | 1 | ? | ? | ? | ? | ? | ? | ? | ? | ? | ? | 1 | ? | 1 | ? | ? | 0 | ? | 1 | 1 | ? | 1 | 1 | 1 | ? | ? | ? | ? | ? | ? | ? | 1 | ? | ? |
| *Thompsonia littoralis* | ? | ? | ? | ? | ? | ? | ? | 1 | ? | ? | 0 | 1 | ? | ? | ? | ? | ? | ? | 1 | 1 | 1 | 1 | 1 | 0 | ? | 1 | 1 | 0 | 1 | 1 | 1 | 1 | ? | ? | 1 | ? | 1 | ? | 1 | ? | ? |
| *Thompsonia magellana* | ? | ? | ? | ? | ? | ? | ? | 1 | ? | ? | ? | 1 | ? | ? | ? | ? | ? | ? | ? | ? | ? | ? | ? | 0 | ? | 1 | ? | ? | 1 | ? | 1 | ? | ? | ? | ? | ? | ? | ? | 1 | ? | ? |
| *Diplothylacus sinensis* | ? | ? | ? | ? | ? | 0 | ? | 1 | ? | ? | 0 | 1 | 1 | ? | ? | 1 | 1 | 0 | 1 | 1 | 1 | 1 | 1 | 0 | ? | 1 | 1 | 0 | 1 | 1 | 1 | ? | 1 | 1 | 1 | 1 | 1 | 1 | 1 | ? | 0 |
| *Polyascus plana* | 0 | 0 | 0 | 0 | 0 | 1 | 1 | 1 | 1 | 1 | 0 | 1 | 1 | ? | 0 | 1 | 1 | 1 | 1 | 1 | 1 | 1 | 1 | 0 | 1 | 1 | 1 | 0 | 1 | 1 | 1 | 1 | 1 | 1 | 1 | ? | 1 | ? | 1 | ? | 0 |
| *Polyascus gregaria* | 0 | 0 | 0 | 0 | 0 | 1 | 1 | 1 | 1 | 1 | 0 | 1 | 1 | ? | ? | 1 | 1 | 1 | 1 | 1 | 1 | 1 | 1 | 0 | 1 | 1 | 1 | 0 | 1 | 1 | 1 | 1 | 1 | 1 | 1 | 1 | ? | ? | 1 | ? | ? |
| *Polyascus polygenea* | 0 | 0 | 0 | 0 | 0 | 1 | 1 | 1 | 1 | 1 | 0 | 1 | 1 | 1 | 0 | 1 | 1 | 1 | 1 | 1 | 1 | 1 | 1 | 0 | 1 | 1 | 1 | 0 | 1 | 1 | 1 | 1 | 1 | 1 | 1 | 1 | 1 | 1 | 1 | ? | ? |
| *Sacculina sinensis* | 0 | 0 | 0 | 0 | 0 | 1 | 1 | 1 | 1 | 1 | 0 | 1 | 1 | ? | ? | 1 | 1 | ? | 1 | 1 | 1 | 1 | 1 | 0 | 1 | 1 | 1 | 0 | 1 | 1 | 1 | ? | ? | ? | 1 | ? | 1 | ? | 1 | ? | ? |
| *Sacculina leptodiae* | ? | ? | ? | ? | ? | ? | ? | ? | ? | ? | ? | ? | ? | ? | ? | ? | ? | ? | ? | ? | ? | ? | ? | 0 | ? | ? | ? | ? | ? | ? | ? | ? | ? | ? | ? | ? | ? | ? | 1 | ? | ? |
| *Sacculina confragosa* | 0 | 0 | 0 | 0 | 0 | 1 | 1 | 1 | 1 | 1 | 0 | 1 | 1 | 1 | 1 | 1 | 1 | 1 | 1 | 1 | 1 | 1 | 1 | 0 | 1 | 1 | 1 | 0 | 1 | 1 | 1 | 1 | 1 | 1 | 1 | 1 | 1 | ? | 1 | ? | ? |
| *Sacculina oblonga* | ? | ? | ? | ? | ? | ? | ? | ? | ? | ? | ? | ? | ? | ? | ? | ? | ? | ? | ? | ? | ? | ? | ? | 0 | ? | ? | ? | ? | ? | ? | ? | ? | ? | ? | ? | ? | ? | ? | 1 | ? | ? |
| *Ibla quadrivalvis* | 0 | 0 | 0 | 0 | 0 | 1 | 1 | 1 | 1 | 0 | 0 | 1 | 1 | 1 | 1 | 1 | 1 | 1 | 1 | 1 | 1 | 1 | 1 | 0 | 1 | 1 | 1 | 0 | 1 | 1 | 1 | 1 | 1 | 1 | 1 | 1 | 1 | 1 | 1 | 0 | 1 |
| *Pollicipes polymerus* | 0 | 0 | 0 | 0 | 0 | 1 | 1 | 1 | 1 | 0 | 1 | 1 | 0 | 1 | 1 | 1 | 1 | 1 | 1 | 1 | 1 | 1 | 1 | 0 | 1 | 1 | 1 | 0 | 1 | 1 | 1 | 1 | 1 | 1 | 1 | ? | 1 | ? | 1 | 1 | 1 |
| *Calantica spinosa* | ? | ? | ? | ? | ? | ? | 1 | ? | ? | ? | ? | ? | ? | ? | ? | ? | ? | ? | ? | ? | ? | ? | ? | 0 | ? | ? | 1 | ? | ? | ? | ? | ? | ? | ? | ? | ? | ? | ? | 1 | 1 | ? |
| *Smilium peronii* | ? | ? | ? | ? | ? | ? | ? | ? | ? | ? | ? | ? | ? | ? | ? | ? | ? | ? | ? | ? | ? | ? | ? | 0 | ? | ? | 1 | ? | ? | ? | ? | ? | ? | ? | ? | ? | ? | ? | 1 | 1 | ? |
| *Capitulum mitella* | 0 | 0 | 0 | 0 | 0 | 1 | 1 | 1 | 1 | 0 | 1 | 1 | 0 | 1 | 1 | 1 | 1 | 1 | 1 | 1 | 1 | 1 | 1 | 0 | 1 | 1 | 1 | 0 | 1 | 1 | 1 | 1 | ? | ? | 1 | 1 | 1 | 1 | 1 | 1 | 1 |
| *Paralepas dannevigi* | ? | ? | ? | ? | ? | 1 | ? | 1 | ? | ? | 0 | 1 | 1 | 1 | 1 | 1 | 1 | ? | 1 | 1 | 1 | 1 | 1 | 0 | 1 | 1 | 1 | 0 | 1 | 1 | 1 | 1 | ? | ? | 1 | 1 | 1 | 1 | 1 | 1 | ? |
| *Conchoderma auritum* | 0 | 0 | 0 | 0 | 0 | 1 | 1 | 1 | 1 | 0 | ? | ? | ? | ? | ? | ? | ? | ? | 1 | 1 | 1 | 1 | 1 | 0 | ? | 1 | 1 | 0 | 1 | 1 | 1 | 1 | ? | ? | 1 | ? | 1 | ? | 1 | 1 | ? |
| *Lepas testudinata* | 0 | 0 | 0 | 0 | 0 | 1 | 1 | 1 | 1 | 0 | 1 | 1 | 1 | 1 | 1 | 1 | 1 | 1 | 1 | 1 | 1 | 1 | 1 | 0 | 1 | 1 | 1 | 0 | 1 | 1 | 1 | 1 | 1 | 1 | 1 | 1 | 1 | 1 | 1 | 1 | 1 |
| *Octolasmis warwickii* | 0 | 0 | 0 | 0 | 0 | 1 | 1 | 1 | 1 | 0 | 0 | 1 | 1 | 1 | 1 | 1 | 1 | 1 | 1 | 1 | 1 | 1 | 1 | 0 | 1 | 1 | 1 | 0 | 1 | 1 | 1 | 1 | 1 | 1 | 1 | 1 | 1 | 1 | 1 | 1 | 1 |
| *Oxynaspis celata* | ? | ? | ? | ? | ? | ? | ? | ? | ? | ? | ? | ? | ? | ? | ? | ? | ? | ? | ? | ? | ? | ? | ? | 0 | ? | ? | 1 | ? | ? | ? | ? | ? | ? | ? | ? | ? | ? | ? | 1 | 1 | ? |
| *Poecilasma inaequilaterale* | ? | ? | ? | ? | ? | ? | ? | ? | ? | ? | ? | ? | ? | ? | ? | ? | ? | ? | ? | ? | ? | ? | ? | 0 | ? | ? | 1 | 0 | 1 | ? | ? | ? | ? | ? | ? | ? | ? | ? | 1 | 1 | ? |
| *Megalasma striatum* | ? | ? | ? | ? | ? | ? | ? | ? | ? | ? | 0 | 1 | 1 | ? | 1 | 1 | 1 | ? | 1 | ? | ? | ? | ? | 0 | ? | 1 | 1 | ? | 1 | 1 | 1 | ? | ? | ? | ? | ? | ? | ? | 1 | 1 | 1 |
| *Ornatoscalpellum stroemi* | ? | ? | ? | ? | ? | 1 | ? | 1 | ? | ? | 0 | 1 | 1 | 1 | 1 | 1 | 1 | 1 | 1 | 1 | 1 | 1 | 1 | 0 | ? | 1 | 1 | 0 | 1 | 1 | 1 | 1 | 1 | 1 | 1 | 1 | 1 | 1 | 1 | 1 | 1 |
| *Trianguloscalpellum regium* | ? | ? | ? | ? | ? | ? | ? | ? | ? | ? | ? | ? | ? | ? | ? | ? | ? | ? | ? | ? | ? | ? | ? | 0 | ? | 1 | 1 | 0 | 1 | ? | ? | ? | ? | ? | 1 | ? | ? | ? | 1 | 1 | ? |
| *Litoscalpellum regina* | ? | ? | ? | ? | ? | ? | ? | ? | ? | ? | ? | ? | ? | ? | ? | ? | ? | ? | 1 | 1 | 1 | ? | ? | 0 | ? | 1 | 1 | 0 | 1 | 1 | 1 | ? | ? | ? | 1 | ? | 1 | ? | 1 | 1 | ? |
| *Scalpellum scalpellum* | 0 | 0 | 0 | 0 | 0 | 1 | 1 | 1 | 1 | 0 | 0 | 1 | 1 | 1 | 1 | 1 | 1 | 1 | 1 | 1 | 1 | 1 | 1 | 0 | 1 | 1 | 1 | 0 | 1 | 1 | 1 | 1 | 1 | 1 | 1 | 1 | 1 | 1 | 1 | 1 | 1 |
| *Neolepas zevinae* | ? | ? | ? | ? | ? | ? | ? | ? | ? | ? | ? | ? | ? | ? | ? | ? | ? | ? | ? | ? | ? | ? | ? | 0 | ? | ? | 1 | ? | ? | ? | ? | ? | ? | ? | ? | ? | ? | ? | 1 | 1 | 1 |
| *Leucolepas longa* | ? | ? | ? | ? | ? | ? | ? | ? | ? | ? | ? | ? | ? | ? | ? | ? | ? | ? | ? | ? | ? | ? | ? | 0 | ? | ? | 1 | ? | ? | ? | ? | ? | ? | ? | ? | ? | ? | ? | 1 | 1 | ? |
| *Vulcanolepas osheai* | ? | ? | ? | ? | ? | ? | ? | ? | ? | ? | ? | ? | ? | ? | ? | ? | ? | ? | ? | ? | ? | ? | ? | 0 | ? | ? | 1 | ? | ? | ? | ? | ? | ? | ? | ? | ? | ? | ? | 1 | 1 | ? |
| *Ashinkailepas seepiophila* | ? | ? | ? | ? | ? | ? | ? | ? | ? | ? | ? | ? | ? | ? | ? | ? | ? | ? | ? | ? | ? | ? | ? | 0 | ? | ? | 1 | ? | ? | ? | ? | ? | ? | ? | ? | ? | ? | ? | 1 | 1 | ? |
| *Neoverruca brachylepadomorfis* | 0 | 0 | 0 | 0 | ? | ? | ? | 1 | ? | ? | ? | ? | ? | ? | ? | ? | ? | ? | 1 | 1 | 1 | ? | 1 | 0 | ? | 1 | 1 | ? | ? | 1 | ? | ? | ? | ? | ? | ? | ? | ? | 1 | 1 | 1 |
| *Lithotrya valentiana* | 0 | 0 | 0 | 0 | 0 | 1 | 1 | 1 | 1 | 0 | ? | ? | ? | ? | ? | ? | ? | 1 | 1 | 1 | 1 | ? | 1 | 0 | ? | 1 | 1 | 0 | 1 | 1 | 1 | 1 | ? | ? | 1 | ? | 1 | ? | 1 | 1 | ? |
| *Verruca stroemia* | 0 | 0 | 0 | 0 | 0 | 1 | 1 | 1 | 1 | 0 | 0 | 1 | 1 | 1 | 1 | 1 | 1 | 1 | 1 | 1 | 1 | 1 | 1 | 0 | 1 | 1 | 1 | 0 | 1 | 1 | 1 | 1 | 1 | 1 | 1 | 1 | 1 | 1 | 1 | 1 | 1 |
| *Metaverruca recta* | ? | ? | ? | ? | ? | ? | ? | ? | ? | ? | ? | ? | ? | ? | ? | ? | ? | ? | ? | ? | ? | ? | ? | 0 | ? | ? | 1 | ? | ? | ? | ? | ? | ? | ? | ? | ? | ? | ? | 1 | 1 | ? |
| *Rostratoverruca kruegeri* | ? | ? | ? | ? | ? | ? | ? | ? | ? | ? | ? | ? | ? | ? | ? | ? | ? | ? | ? | ? | ? | ? | ? | 0 | ? | ? | 1 | ? | ? | ? | ? | ? | ? | ? | ? | ? | ? | ? | 1 | 1 | ? |
| *Catomerus polymerus* | ? | ? | ? | ? | ? | ? | ? | 1 | ? | ? | ? | ? | ? | ? | ? | ? | ? | ? | ? | ? | ? | ? | ? | 0 | ? | 1 | 1 | ? | ? | ? | ? | ? | ? | ? | ? | ? | ? | ? | 1 | 1 | ? |
| *Chamaesipho tasmanica* | 0 | 0 | 0 | 0 | 0 | 1 | 1 | 1 | 1 | 0 | ? | ? | ? | ? | ? | ? | ? | 1 | 1 | 1 | ? | ? | ? | 0 | ? | 1 | 1 | 0 | 1 | 1 | 1 | 1 | ? | ? | 1 | ? | 1 | ? | 1 | 1 | ? |
| *Notochthamalus scabrosus* | ? | ? | ? | ? | ? | ? | ? | ? | ? | ? | ? | ? | ? | ? | ? | ? | ? | ? | ? | ? | ? | ? | ? | 0 | ? | ? | 1 | ? | 1 | ? | 1 | ? | ? | ? | 1 | ? | ? | ? | 1 | 1 | ? |
| *Chthamalus montagui* | 0 | 0 | 0 | 0 | 0 | 1 | 1 | 1 | 1 | 0 | 0 | 1 | 1 | 1 | 1 | 1 | 1 | 1 | 1 | 1 | 1 | 1 | ? | 0 | 1 | 1 | 1 | 0 | 1 | 1 | 1 | 1 | ? | ? | 1 | 1 | 1 | 1 | 1 | 1 | ? |
| *Jehlius cirratus* | ? | ? | ? | ? | ? | ? | ? | ? | ? | ? | ? | ? | ? | ? | ? | ? | ? | ? | ? | ? | ? | ? | ? | 0 | ? | ? | 1 | ? | ? | ? | ? | ? | ? | ? | ? | ? | ? | ? | 1 | 1 | ? |
| *Elminius kingii* | 0 | 0 | 0 | 0 | 0 | 1 | 1 | 1 | 1 | 0 | 0 | 1 | 1 | 1 | 1 | 1 | 1 | 1 | 1 | 1 | 1 | 1 | 1 | 0 | 1 | 1 | 1 | 0 | 1 | 1 | 1 | 1 | 1 | 1 | 1 | 1 | 1 | 1 | 1 | 1 | 1 |
| *Tetraclitella divisa* | 0 | 0 | 0 | 0 | 0 | 1 | 1 | 1 | 1 | 0 | ? | 1 | ? | ? | ? | ? | ? | 1 | 1 | 1 | 1 | ? | ? | 0 | 1 | 1 | 1 | 0 | 1 | 1 | 1 | 1 | 1 | 1 | 1 | 1 | 1 | 1 | 1 | 1 | ? |
| *Tetraclita japonica* | 0 | 0 | 0 | 0 | 0 | 1 | 1 | 1 | 1 | 0 | 0 | 1 | 1 | 1 | 1 | 1 | 1 | 1 | 1 | 1 | 1 | 1 | 1 | 0 | 1 | 1 | 1 | 0 | 1 | 1 | 1 | 1 | 1 | 1 | 1 | 1 | 1 | 1 | 1 | 1 | ? |
| *Chelonibia patula* | 0 | 0 | 0 | 0 | 0 | 1 | 1 | 1 | 1 | 0 | ? | ? | ? | ? | ? | ? | ? | 1 | 1 | 1 | 1 | ? | ? | 0 | ? | 1 | 1 | 0 | 1 | 1 | 1 | 1 | ? | ? | 1 | ? | 1 | ? | 1 | 1 | ? |
| *Semibalanus cariosus* | 0 | 0 | 0 | 0 | 0 | 1 | 1 | 1 | 1 | 0 | 0 | 1 | 1 | 1 | 1 | 1 | 1 | 1 | 1 | 1 | 1 | 1 | 1 | 0 | 1 | 1 | 1 | 0 | 1 | 1 | 1 | 1 | 1 | 1 | 1 | 1 | 1 | 1 | 1 | 1 | 1 |
| *Balanus balanus* | 0 | 0 | 0 | 0 | 0 | 1 | 1 | 1 | 1 | 0 | 0 | 1 | 1 | 1 | 1 | 1 | 1 | 1 | 1 | 1 | 1 | 1 | 1 | 0 | 1 | 1 | 1 | 0 | 1 | 1 | 1 | 1 | 1 | 1 | 1 | 1 | 1 | 1 | 1 | 1 | ? |
| *Menesiniella aquila* | ? | ? | ? | ? | ? | ? | ? | ? | ? | ? | ? | ? | ? | ? | ? | ? | ? | ? | ? | ? | ? | ? | ? | 0 | ? | ? | 1 | ? | ? | ? | ? | ? | ? | ? | ? | ? | ? | ? | 1 | 1 | ? |
| *Megabalanus tintinnabulum* | 0 | 0 | 0 | 0 | 0 | 1 | 1 | 1 | 1 | 0 | 0 | 1 | 1 | 1 | 1 | 1 | 1 | 1 | 1 | 1 | 1 | 1 | 1 | 0 | ? | 1 | 1 | 0 | 1 | 1 | 1 | 1 | 1 | 1 | 1 | 1 | 1 | 1 | 1 | 1 | ? |
| *Austromegabalanus psittacus* | 0 | 0 | 0 | 0 | 0 | 1 | 1 | 1 | 1 | 0 | ? | ? | ? | ? | ? | ? | ? | 1 | 1 | ? | ? | ? | ? | 0 | ? | 1 | 1 | 0 | 1 | 1 | 1 | 1 | ? | ? | 1 | ? | 1 | ? | 1 | 1 | ? |
